# Mitigating drought stress and enhancing maize resistance through biopriming with *Rhizopus arrhizus*: insights into Morpho-Biochemical and molecular adjustments

**DOI:** 10.1186/s12870-025-06793-3

**Published:** 2025-06-11

**Authors:** Asmaa S. Taha, Hoda A. Fathey, Asmaa H. Mohamed, Amira A. Ibrahim, Mohamed Abdel-Haleem

**Affiliations:** 1 Department of Botany and Microbiology, Faculty of Science, Zagazig University, Zagazig, 44519 Egypt; 2 Department of Botany and Microbiology Faculty of Science, Arish University, Arish, Egypt

**Keywords:** *Zea mays*, Biopriming, Drought stress, *Rhizopus arrhizus*, ISSR fingerprinting

## Abstract

**Background:**

Drought stress represents a significant threat to crop productivity, particularly in regions characterized by water scarcity. This study investigated the potential of utilizing endophytic fungi to enhance drought tolerance in maize (*Zea mays* L.). Specifically, we aimed to investigate the role of these fungi in improving the physiological, morphological, and molecular responses of maize plants subjected to drought conditions.

**Results:**

Our findings revealed a significant contribution of endophytic fungi in mitigating the adverse effects of drought stress. Morphological analysis revealed higher root and shoot growth in treated plants compared to untreated controls, indicating improved water uptake and retention capabilities. Furthermore, physiological parameters, including chlorophyll content, markedly increased in fungus-treated plants under drought conditions. The activities of enzymatic antioxidants, including catalase (CAT), peroxidase (POX), and polyphenol oxidase (PPO), in maize plants inoculated with *R. arrhizus* under severe drought stress conditions were increased by 157.71%, 92.14%, and 144.44%, respectively, compared to those of the non-bioprimed plants. Endophytic inoculation resulted in a reduction of H₂O₂ and MDA levels by 48% and 55.11%, respectively, compared to non-inoculated plants. At the molecular level, ISSR analysis revealed distinct banding patterns between inoculated and non-inoculated plants under drought stress, indicating genomic variation linked to the presence of endophytic fungi. These molecular fingerprints suggest the activation of stress-responsive pathways and highlight the potential role of endophytes in enhancing plant drought tolerance. Collectively, these results highlight the potential of utilizing endophytic fungi as a sustainable and eco-friendly approach to enhance drought tolerance in maize, offering promising implications for agricultural practices in arid and semiarid regions.

**Conclusions:**

This study represents one of the few investigations detailing the practical application of endophytic fungi-especially *Rhizopus arrhizus*, in mitigating the detrimental effects of drought stress caused by limited water availability. These findings raise the possibility of utilizing endophytes suited to dry environments within agricultural systems.

**Supplementary Information:**

The online version contains supplementary material available at 10.1186/s12870-025-06793-3.

## Introduction

Maize (*Zea mays* L.) ranks among the three primary food crops alongside wheat and rice [1] and is a significant resource for both feed and industrial applications [2]. Global climate change and increasing temperatures are projected to induce geographical variations in rainfall patterns, thereby increasing the frequency and severity of drought conditions faced by cultivated crops [3]. Given this context, maize production is already restricted by drought [4], with estimates indicating a reduction of 25–30% in yields within certain areas [5]. Prior research has demonstrated that drought intensity and growth stage significantly impact *Z. mays* productivity [6], while seedling-stage exposure to drought reduces total plant biomass [7]. The morphological characteristics of *Z. mays* are significantly influenced by drought exposure, particularly from the shoot elongation phase (jointing stage) through the early grain filling stage (milk phase). Notably, identical drought levels reduce photosynthetic rates more severely at the tasseling stage than at earlier developmental stages [8]. A variety of investigations have examined the influence of drought on physiological processes [9, 10], which include adverse effects on ion uptake and ionic balance, respiration, photosynthesis, enzyme production, stem and root growth, and solute accumulation. The intensity of these impacts is determined by both drought duration and the organism’s growth stage, representing a major ecological stress [11].

The use of chemical remedies to reduce drought stress may have negative environmental consequences; therefore, microbes offer a sustainable alternative [12, 13]. Fungal endophytes- often bacteria or fungi residing within plant cells without harming the host – play a crucial role in this process [14]. A diverse collection of asymptomatic fungi, known as fungal endophytes, can invade plant tissue, interacting with their host through saprophytic, commensal, or mutualistic relationships [15]. Fungal endophytes increase drought tolerance in host plants by improving water uptake and retention via various mechanisms [4]. Fungal filament networks infiltrate the soil, accessing water reserves inaccessible to plant roots, thereby ensuring a sufficient water supply during dry periods [16]. Like other abiotic stressors, drought induces the production of reactive oxygen species (ROS) that accumulate within plant cells, causing oxidative stress. Fungal endophytes mitigate this oxidative stress by stimulating host plants’ antioxidant defense mechanisms; for example, certain fungi produce superoxide dismutase (SOD) and catalase (CAT), which scavenge ROS that protect plant cells from oxidative damage [17, 18]. Furthermore, under dry conditions, endophytic fungi promote the accumulation of proline, an osmoprotectant that maintains cellular equilibrium [19]. Drought-induced stomatal closure, resulting in chloroplast dehydration and reduced CO_2_ diffusion, suppresses photosynthetic efficiency [20]. Inoculation with endophytic fungi has been shown to stimulate chlorophyll biosynthesis, as demonstrated in aromatic rice [21], wheat plants [22], and maize plants [23].

In addition, drought conditions inhibit carbohydrate metabolism, suppressing plant growth [24]. Ozturk et al. [25] reported that plants mitigate the adverse effects of drought stress by increasing their cellular osmotic potential and accumulating soluble sugars. Endophytic fungal inoculation enhances the production of plant osmolytes, thereby decreasing water loss, preventing chloroplast dehydration, and stimulating plant growth under severe conditions [26–28]. Drought stress also results in the accumulation of ROS, which increases the degree of oxidative damage to macromolecules such as lipids, proteins, DNA, and carbohydrates [29]. Plants subjected to water deficiency with endophytic fungi exhibited increased production of phenolic compounds. These phenolic compounds significantly minimize the harmful impacts of stress on plant growth by scavenging ROS and protecting membranes from damage [30].

Owing to improvements in gene sequencing methods and the utilization of genetic markers, the amount of genomic data available for agricultural and medicinal plant species has increased dramatically over the last ten years [31]. Compared with other techniques, inter simple sequence repeats (ISSRs) have demonstrated significant success in identifying a notably greater number of polymorphic fragments per primer [32]. Molecular markers are invaluable instruments in plant breeding since they are unaffected by environmental factors, which is a significant advantage of this methodology. Specifically, ISSR is a potent technology capable of accurately and efficiently identifying a wide variety of polymorphic DNA fragments, thereby driving the development of DNA sequencing and plant genomic research [33, 34].

*Rhizopus* species are filamentous fungi commonly found in soil, decaying fruits, vegetables, and food products [35]. Several studies have highlighted the ability of *Rhizopus arrhizus* to produce metabolites that regulate plant growth [36, 37]. *Rhizopus* sp. are recognized as plant growth-promoting fungi (PGPF), known to enhance plant development and improve tolerance to various stress conditions [38]. As endophytes, *Rhizopus* sp. contribute to plant stress resilience through various mechanisms, including secreting bioactive compounds [39], modulating hormonal balance [40], and enhancing nutrient solubilization and uptake [41].

Numerous studies have examined *Z. mays* physiological and biochemical responses to drought stress, but few (to our knowledge) have examined its genomic changes using molecular markers like ISSR, especially in combination with beneficial microbial inoculants like *Rhizopus* sp. Therefore, this study provides molecular-level insights into DNA stability and genetic polymorphism under drought conditions, demonstrating that *Rhizopus* treatment can reduce genomic instability, hence serving as a biotechnological strategy for enhancing plant resilience.

This study aimed to evaluate the effect of biopriming *Z. mays* with the endophytic fungus *Rhizopus arrhizus* under drought stress. The work focused on assessing plant responses at the morphological, biochemical, and genetical levels. Morphological traits included shoot and root length, as well as fresh and dry weights. Biochemical analyses involved measuring proline content, hydrogen peroxide (H₂O₂), malondialdehyde levels (lipid peroxidation), and the activities of antioxidant enzymes catalase (CAT), peroxidase (POX), and polyphenol oxidase (PPO). The study also quantified photosynthetic pigments (chlorophyll a, chlorophyll b, and carotenoids), carbohydrate content, total phenolics, and flavonoid levels. ISSR markers were used at the molecular level to assess genetic variation and genomic template stability (GTS%) in *Zea mays* under drought stress and fungal biopriming. Analysis included polymorphic banding patterns and genetic relationships among treatments.

## Results

### **Isolation of endophytic fungi from*Z. mays*plants**

**Fig. 1:**
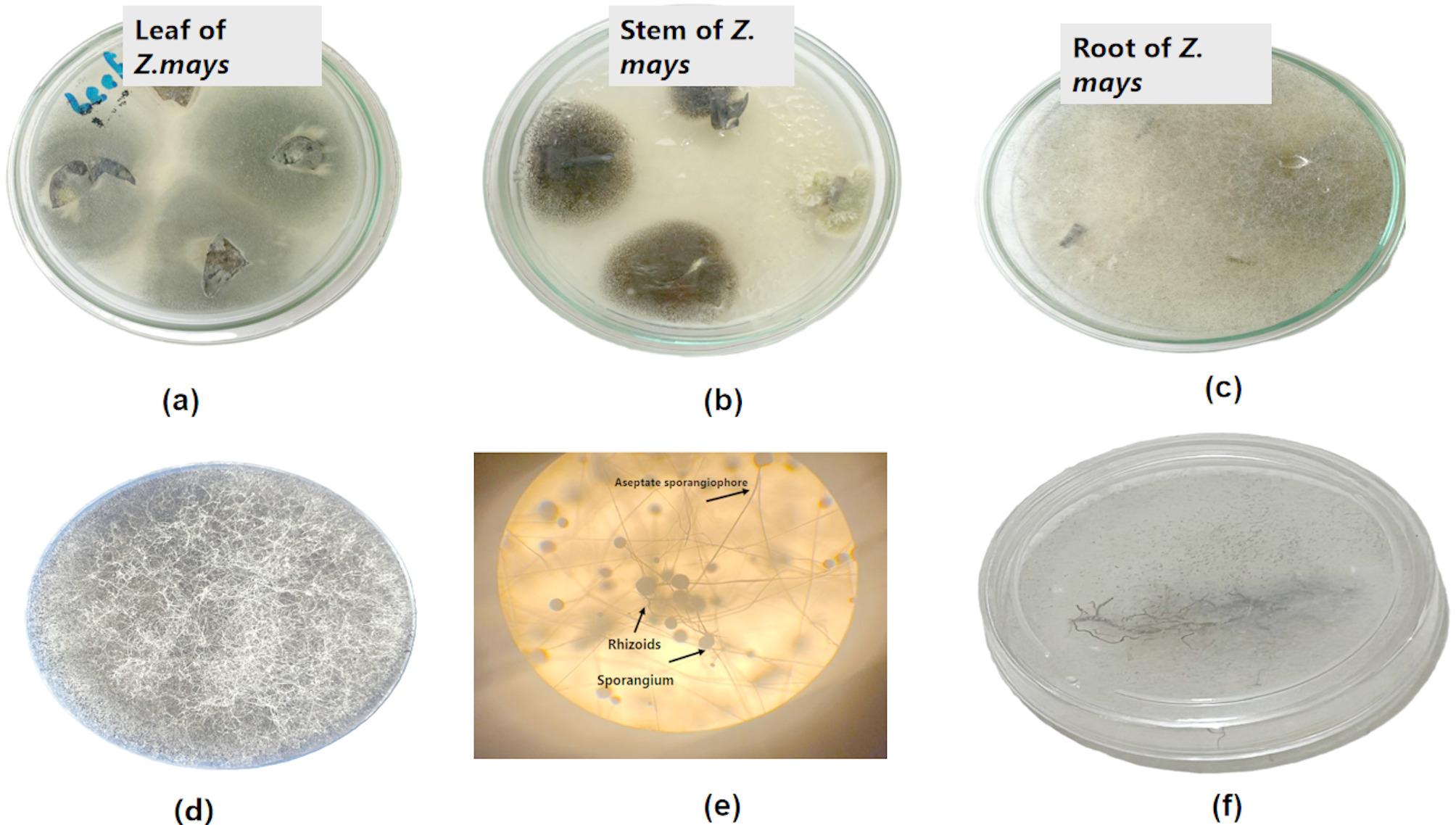
Isolation of endophytic fungi from *Z. mays* **(a)** leaves, **(b)** stem, and **(c)** roots. **(d)** the growth of *Rhizopus* on potato dextrose agar (PDA) medium, **(e**) photograph of *Rhizopus* under a light microscope showing sporangium, aseptate sporangiophore, and rhizoids, and **(f)** plating long segments of *Z. mays* root on a PDA plate confirming colonization of roots by *Rhizopus*

**Fig. 2:**
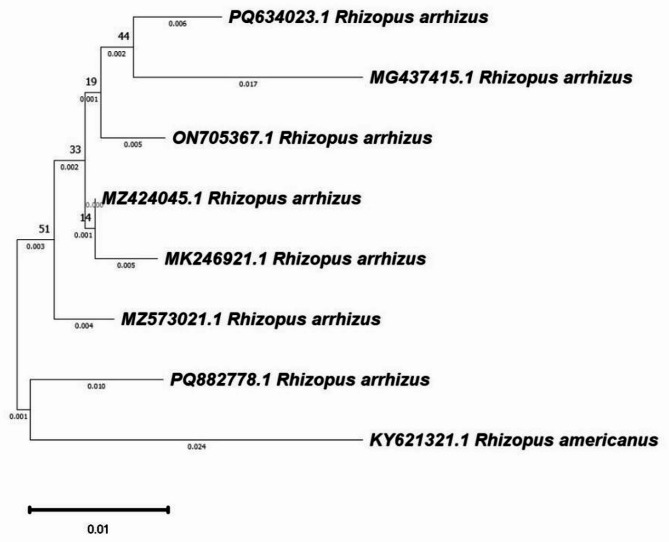
Phylogenetic analysis of *Rhizopus arrhizus* (acc no. PQ882778) demonstrating the ITS connection with closely similar strains’ ITS sequences that were retrieved from NCBI GenBank database. In the MEGA7 software, evolutionary analyses were carried out

In the current study, endophytic fungi were isolated from different plant parts (roots, stems, and leaves) of *Z. mays* plants (Fig. 1a, b, and c). Initial identification of these fungi was conducted using universal keys on the basis of their visual characteristics, revealing that they belong to the *Rhizopus* and *Aspergillus* genera. Specifically, *Rhizopus arrhizus* was selected due to limited knowledge regarding its potential role on plants under stress conditions, such as drought. The selected fungal endophyte was morphologically identified, with macroscopic colony features confirmed through comparison of agar plate observations with microscopic features under a light microscope. Colonies of *Rhizopus* on potato dextrose agar (PDA) medium grew rapidly, resulting in white, cottony formations that then converted from brownish gray to blackish gray as sporulation progressed (Fig. 1d). Well-developed rhizoids were observed in opposition to sporangiophores under a light microscope (Fig. 1e). Furthermore, significant sequence similarities (99–100%) between our isolate and related isolates were found via molecular analysis. In accordance with the NCBI database records, *Rhizopus arrhizus* was added to GenBank with accession number PQ882778 (Fig. 2).

### **Root colonization by*Rhizopus***

The endophytic fungus *R. arrhizus* successfully colonized the roots of maize, improving its resistance to drought, as confirmed by PDA culture. All *Rhizopus*-treated *Zea mays* showed nonseptate mycelial outgrowth with sporangia and rhizoids in contrast to the sporangiophores observed on their sections, representing 100% fungal colonization (Fig. 1f).

### **Morphological and physiological responses of*Z. mays*under drought conditions**

#### **Effect of drought and the application of***R. arrhizus****R. arrhizus*on morphological parameters of*Z. mays*plants**

The treatments included: T1– control (800 mL H₂O); T2– control with *R. arrhizus* (800 mL H₂O); T3– mild drought stress (400 mL H₂O); T4– mild drought stress with *R. arrhizus* (400 mL H₂O); T5– severe drought stress (200 mL H₂O); and T6– severe drought stress with *R. arrhizus* (200 mL H₂O), as shown in Table 1. Water deficiency generally suppressed plant growth parameters; however, inoculation with *R. arrhizus* partially alleviated these adverse effects, as shown in Table 2. Under well-watered conditions, fungal inoculation resulted in the greatest enhancement of shoot, root, and leaf lengths, reaching 40 ± 2.116 cm, 27 ± 1.428 cm, and 28 ± 1.481 cm, respectively, compared to non-inoculated plants. Under severe drought conditions (200 mL irrigation), the most pronounced reductions were observed. In non-inoculated plants, shoot, root, and leaf lengths declined to 25 ± 1.322 cm, 18 ± 0.952 cm, and 19 ± 1.005 cm, respectively. In contrast, inoculated plants under the same stress level maintained comparatively greater lengths: 35 ± 1.852 cm for shoots, 26 ± 1.375 cm for roots, and 22 ± 1.481 cm for leaves.

**Table 1:** Different treatments

T1	T2	T3	T4	T5	T6
Control (800 mL H_2_O)	Control + *Rhizopus*	400 mL H_2_O	400 mL H_2_O + *Rhizopus*	200 mL H_2_O	200 mL H_2_O + *Rhizopus*

**Table 2:** Effect of drought and the application of *R. arrhizus* on morphological parameters of *Z. mays* plants

Parameters Treatments	shoot length (cm)	Root length (cm)	leaf length (cm)	FW shoot (g)	FW root (g)	DW shoot (g)	DW root (g)
**T1**	26 ± 1.375c	12 ± 0.634c	22 ± 1.164b	2.06 ± 0.108 cd	1.95 ± 0.103c	0.274 ± 0.014a	0.193 ± 0.102c
**T2**	40 ± 2.116a	27 ± 1.428a	28 ± 1.481a	4.53 ± 0.239a	2.49 ± 0.131b	0.511 ± 0.027a	0.270 ± 0.014b
**T3**	30 ± 1.587bc	17 ± 0.899b	20 ± 1.058b	2.18 ± 0.108c	1.76 ± 0.093c	0.369 ± 0.019b	0.222 ± 0.011c
**T4**	36 ± 1.904a	26 ± 1.375a	27 ± 1.428a	2.93 ± 0.154b	3.35 ± 0.177a	0.502 ± 0.026a	0.418 ± 0.022a
**T5**	25 ± 1.322c	18 ± 0.952b	19 ± 1.005b	1.61 ± 0.084d	1.79 ± 0.094c	0.297 ± 0.015c	0.179 ± 0.009c
**T6**	35 ± 1.852ab	26 ± 1.375a	22 ± 1.481a	2.36 ± 0.124c	2.03 ± 0.107c	0.413 ± 0.021b	0.270 ± 0.014b

Data are the mean (± SE) for *n* = 3. One-Way ANOVA: Duncan multiple range test (DMRT) finds no significant difference (*p* < 0.05) between the means of the same column accompanied by the same letter. Treatments: T1-control (800 mL H₂O); T2– control with *Rhizopus*; T3– 400 mL H₂O; T4– 400 mL H₂O with *Rhizopus*; T5– 200 mL H₂O; T6– 200 mL H₂O with *Rhizopus*

Also, plants inoculated with *R. arrhizus* under normal irrigation exhibited significantly higher fresh and dry shoot biomass (4.53 ± 0.239 g and 0.511 ± 0.027 g, respectively), while mild drought (400 mL) irrigation showed a relatively high value (2.93 ± 0.154 and 0.502 ± 0.026, respectively) (Table 3). In contrast, the lowest shoot fresh weight was recorded under severe drought stress in non-inoculated plants (1.61 ± 0.084 g). However, the minimum shoot dry weight was observed in untreated plants grown under well-watered conditions (0.274 ± 0.014 g). The highest fresh and dry root biomass was significantly recorded in inoculated plants under mild drought stress, reaching 3.35 ± 0.177 g and 0.418 ± 0.022 g, respectively. In contrast, the lowest fresh root weights were observed in non-inoculated plants subjected to mild (1.76 ± 0.093 g) and severe (1.79 ± 0.094 g) drought stress. The minimum dry root weight (0.179 ± 0.009 g) was detected in plants exposed to severe drought without fungal inoculation.

**Table 3:** Effect of inoculating *R. arrhizus* on pigment fractions (mg/g FW) of *Z. mays* plants under drought stress

Parameters Treatments	Chl a (mg/g FW)	Chl b (mg/g FW)	Carotenoids (mg/g FW)
**T1**	1.175 ± 0.058b	0.533 ± 0.098b	0.333 ± 0.066a
**T2**	1.214 ± 0.079a	0.654 ± 0.058a	0.291 ± 0.084b
**T3**	1.077 ± 0.0586d	0.440 ± 0.057d	0.285 ± 0.077b
**T4**	1.09 ± 0.0579c	0.490 ± 0.059c	0.216 ± 0.092d
**T5**	0.977 ± 0.0621e	0.361 ± 0.0821e	0.241 ± 0.102c
**T6**	1.07 ± 0.107d	0.371 ± 0.1077e	0.219 ± 0.11d

Data are the mean (± SE) for *n* = 3. One-Way ANOVA: Duncan multiple range test (DMRT) finds no significant difference (*p* < 0.05) between the means of the same column accompanied by the same letter. Treatments: T1-control (800 mL H₂O); T2– control with *Rhizopus*; T3– 400 mL H₂O; T4– 400 mL H₂O with *Rhizopus*; T5– 200 mL H₂O; T6– 200 mL H₂O with *Rhizopus*

### **Enzymatic antioxidants**

**Fig. 3:**
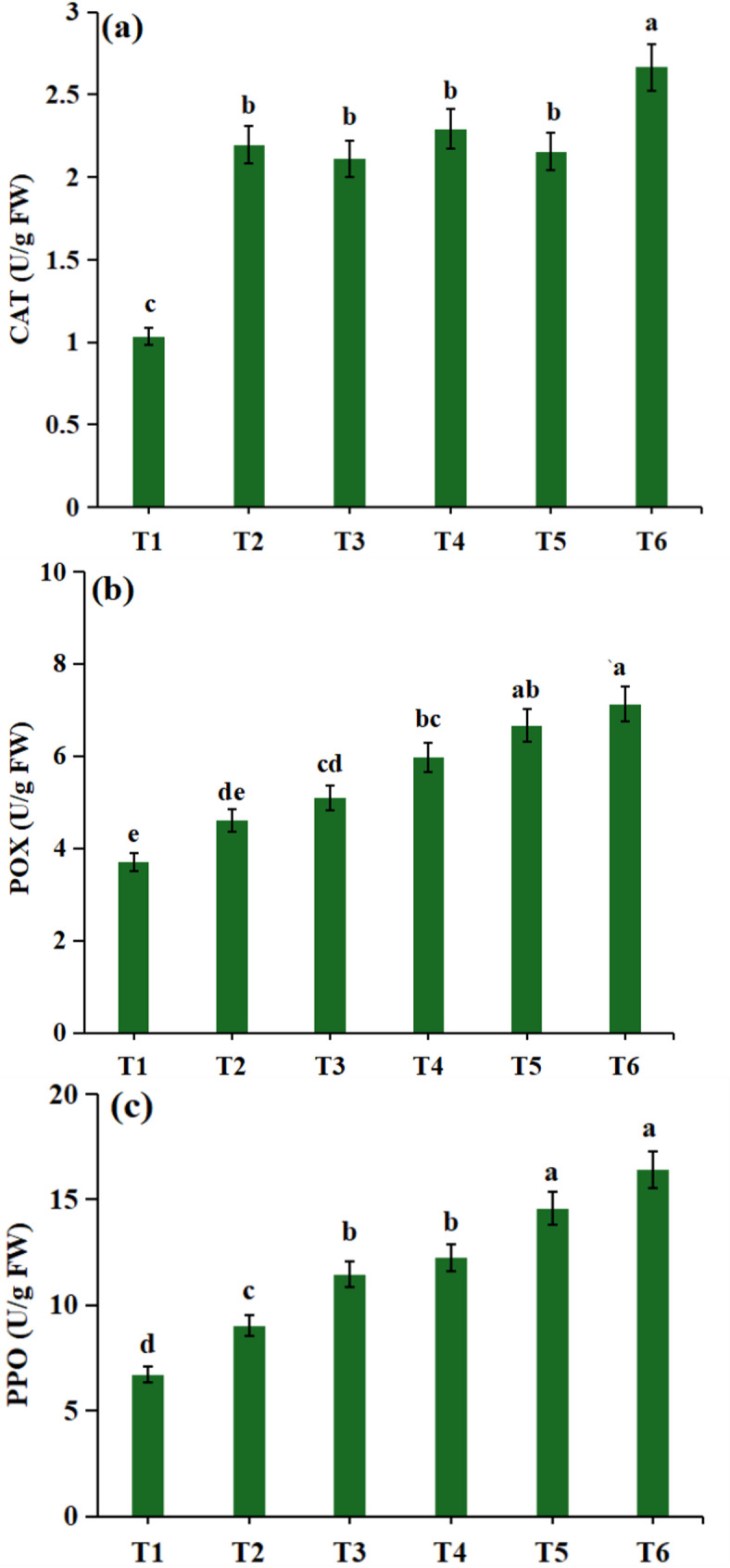
Effect of *Rhizopus* application on antioxidant enzymes system: **(a)** CAT, **(b)** POX, and **(c)** PPO in *Zea mays* plants in response to drought stress. Results are the mean of three replicates±SE. Different letters above bars indicate a significant difference between treatments using One-Way ANOVA followed by Duncan’s multiple range test (DMRT) (*p* < 0.05).Treatments: **T1** -control (800 mL H₂O); **T2**– control with *Rhizopus*; **T3**– 400 mL H₂O; **T4**– 400 mL H₂O with *Rhizopus*; **T5**– 200 mL H₂O; **T6**– 200 mL H₂O with *Rhizopus*

CAT, POX, and PPO are essential antioxidant enzymes that play crucial roles in increasing the ability of plants to acclimatize and ultimately survive under drought stress conditions. As shown in Fig. 3, the activities of these enzymes significantly increased in response to drought stress in *Zea mays* plants. Severe drought stress dramatically increased the activities of CAT, POX, and PPO by 108.33%, 79.47%, and 117.16%, respectively, in comparison with those in plants cultivated under controlled conditions. However, when *Zea mays* was treated with *R. arrhizus* under severe drought stress conditions, the activities of these enzymes further increased by 157.71%, 92.14%, and 144.44%, respectively, compared to those of the non-bioprimed plants. These results underscore the role of *Rhizopus* in enhancing the antioxidant defense system of *Zea mays* in response to drought stress.

### **Measurement of proline and total soluble protein contents**

**Fig. 4:**
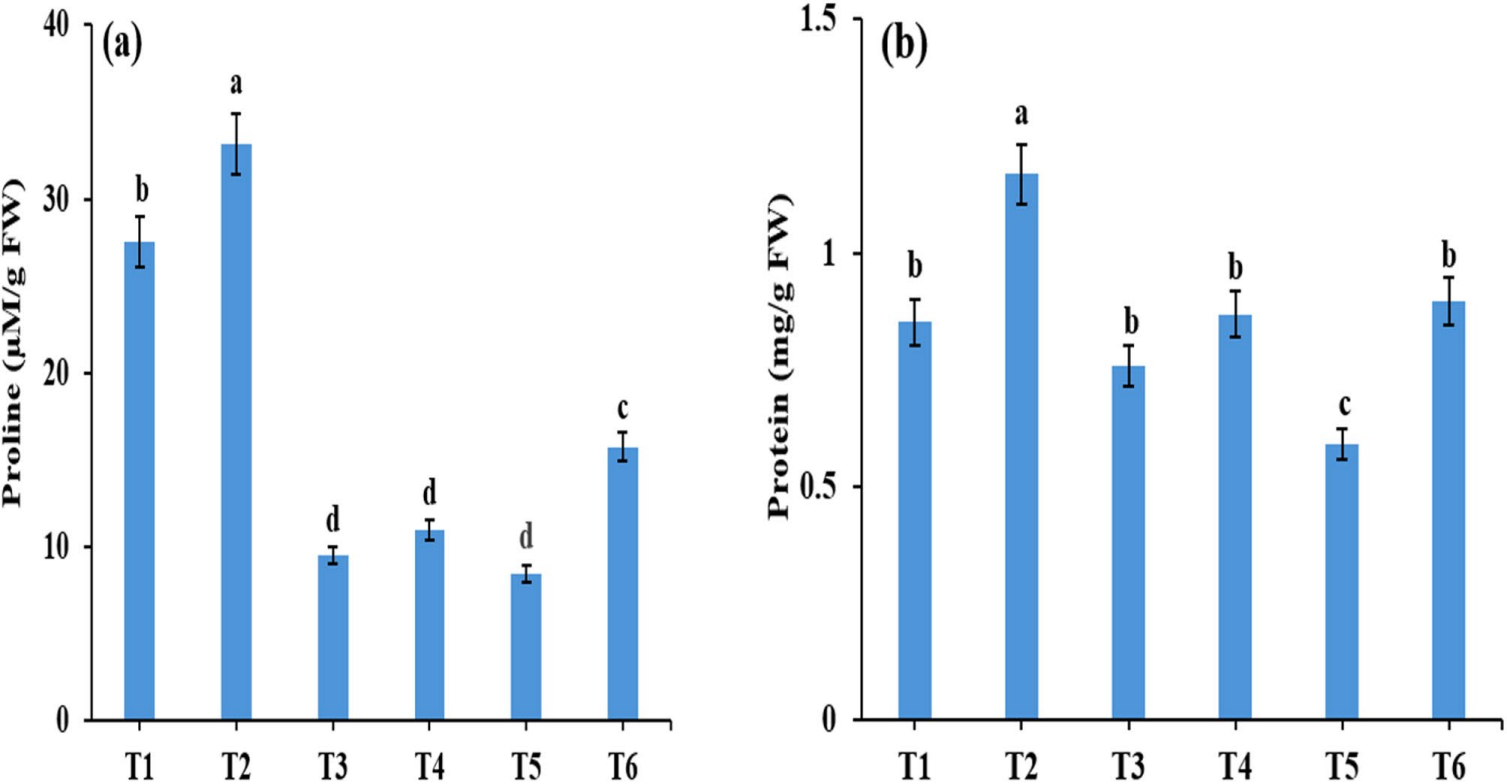
Effect of *Rhizopus* application on **(a)** proline and **(b)** total soluble protein in *Zea mays* plants in response to drought stress. Results are the mean of three replicates±SE. Different letters above bars indicate a significant difference between treatments using One-Way ANOVA followed by Duncan’s multiple range test (DMRT) (*p* < 0.05).Treatments: **T1**-control (800 mL H₂O); **T2**– control with *Rhizopus*; **T3**– 400 mL H₂O; **T4**– 400 mL H₂O with *Rhizopus*; **T5**– 200 mL H₂O; **T6**– 200 mL H₂O with *Rhizopus*

In the present study, the levels of cellular osmolytes were significantly influenced by drought stress and the application of *Rhizopus*, as demonstrated in Fig. 4. The contents of proline and total soluble protein exhibited distinct patterns, with drought stress leading to a reduction in their levels in the leaves of *Zea mays* compared with plants grown under well-watered conditions. However, under both well-watered and drought conditions, the application of *R. arrhizus* effectively increased the proline and total soluble protein contents compared with those of non-bioprimed plants. Specifically, under severe drought stress, compared with non-bioprimed plants, *Zea mays* leaves treated with *R. arrhizus* presented an 86.10% increase in proline content and a 51.58% increase in total soluble protein content, indicating the beneficial effects of *Rhizopus* biopriming in mitigating drought-induced osmotic stress.

### **Influence of*R. arrhizus*on H₂O₂ and MDA contents in*Z. mays*under drought**

**Fig. 5:**
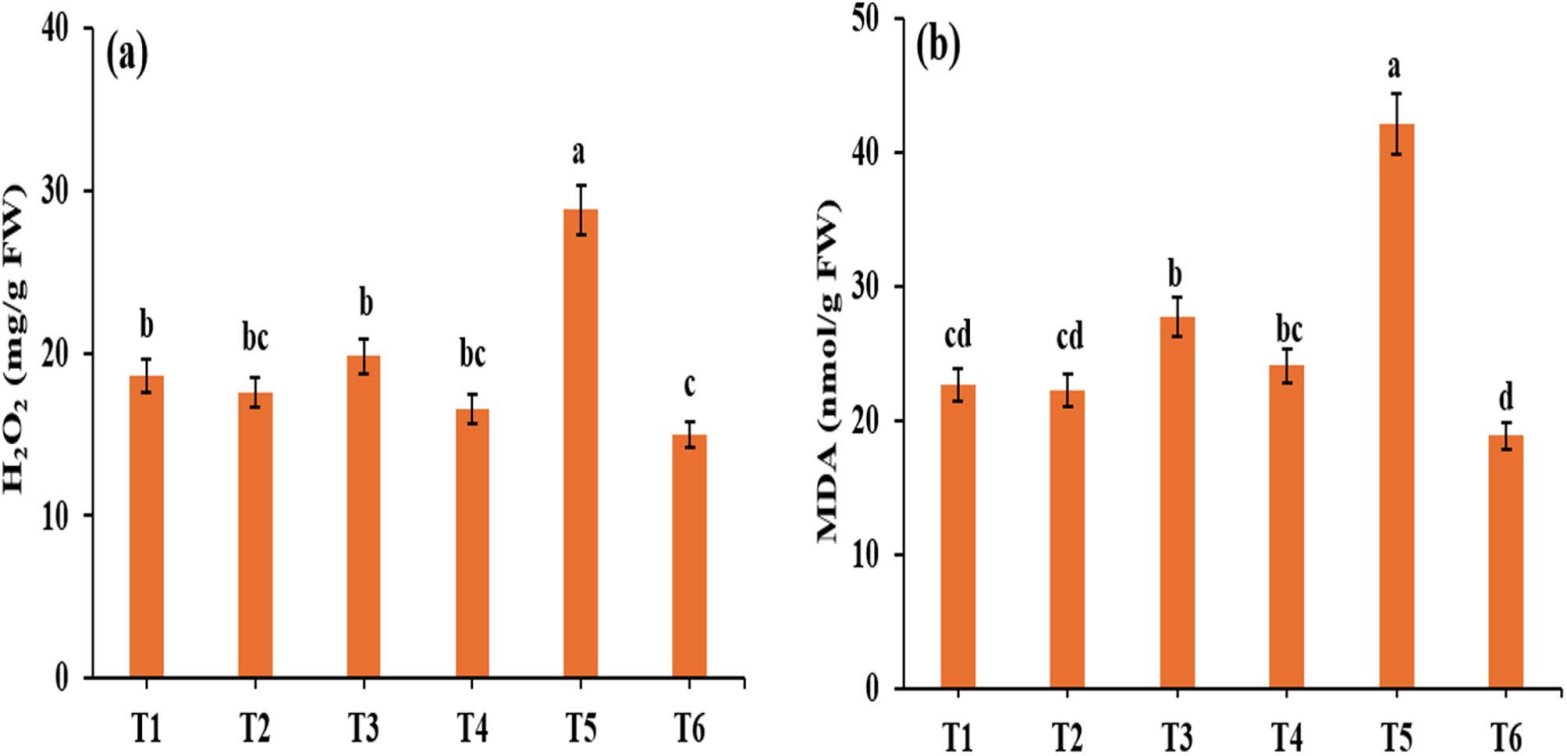
Effect of *Rhizopus* application on stress markers: **(a)** H_2_O_2_ and **(b)** malondialdehyde (MDA) in *Zea mays* plants in response to drought stress. Results are the mean of three replicates±SE. Different letters above bars indicate a significant difference between treatments using One-Way ANOVA followed by Duncan’s multiple range test (DMRT) (*p* < 0.05). Treatments: **T1**-control (800 mL H₂O); **T2**– control with *Rhizopus*; **T3**– 400 mL H₂O; **T4**– 400 mL H₂O with *Rhizopus*; **T5**– 200 mL H₂O; **T6**– 200 mL H₂O with *Rhizopus*

This study also evaluated the oxidative stress markers caused by ROS generation in *Zea mays* under drought, which leads to lipid peroxidation. The contents of H₂O₂ and MDA were measured, as shown in Fig. 5. The results indicated that moderate (T3) and severe (T5) drought stress significantly increased the production of H₂O₂ (19.8 and 28.86 mg/g FW, respectively) and MDA (27.78 and 42.15 nmol/g FW, respectively) compared with that in control plants (T1), which presented lower levels of H₂O₂ (18.60 mg/g FW) and MDA (22.7 nmol/g FW). Conversely, compared with non-biopriming, inoculation with *R. arrhizus* under severe drought conditions reduced the contents of H₂O₂ and MDA by 48% and 55.11%, respectively. The positive impact of *R. arrhizus* was evident in its ability to lower H₂O₂ and MDA levels under both well-watered and drought-stressed conditions, as illustrated in Fig. 5. These findings suggest that *R. arrhizus* plays a vital role in reducing ROS accumulation and alleviating oxidative stress in *Zea mays*, thereby increasing its tolerance to drought.

### **Influence of*R. arrhizus*on photosynthetic pigments in*Z. mays*under drought stress**

The chlorophyll a, chlorophyll b, and carotenoid contents of the growing *Zea mays* plants under drought stress are presented in Table 4. Compared with non-inoculated plants, the inoculation of maize with *R. arrhizus* significantly stimulated the synthesis of chlorophyll a and chlorophyll b under either normal or drought conditions. The maximum value of the photosynthetic pigments was obtained by inoculating *R. arrhizus* with the tested plant (chlorophyll a, 1.21 ± 0.079 chlorophyll b, 0.65 ± 0.058 mg/g FW) under normal conditions compared with the control (non-inoculated) (chlorophyll a, 1.18 ± 0.058 and chlorophyll b, 0.53 ± 0.098 mg/g FW). The synthesis of chlorophyll a, chlorophyll b, and carotenoids is inversely proportional to increasing drought conditions. As a result, the lowest contents of chlorophyll a and chlorophyll b (0.97 ± 0.062 and 0.36 ± 0.082 mg/g FW, respectively) were obtained at the highest drought level.

**Table 4:** Effect of inoculating *R. arrhizus* on carbohydrates contents (mg/g DW) of *Z. mays* plants under drought stress

Parameters Treatments	Souble sugars (mg/g DW)	Insoluble sugars (mg/g DW)	Total carbohydrates (mg/g DW)
**T1**	24.18 ± 0.78f	33.48 ± 0.65a	57.66 ± 0.78c
**T2**	38.96 ± 0.57b	25 ± 0.38b	63.96 ± 0.69a
**T3**	35.23 ± 0.65d	15.02 ± 0.97d	50.25 ± 0.71e
**T4**	41.43 ± 0.74a	17.04 ± 0.82c	58.47 ± 0.63b
**T5**	36.9 ± 0.36c	8.64 ± 0.74f	45.54 ± 0.74f
**T6**	29.85 ± 0.57e	12.18 ± 0.63e	54.21 ± 0.92d

Data are the mean (± SE) for *n* = 3. One-Way ANOVA: Duncan multiple range test (DMRT) finds no significant difference (*p* < 0.05) between the means of the same column accompanied by the same letter.Treatments: T1-control (800 mL H₂O); T2– control with *Rhizopus*; T3– 400 mL H₂O; T4– 400 mL H₂O with *Rhizopus*; T5– 200 mL H₂O; T6– 200 mL H₂O with *Rhizopus*

Conversely, the carotenoid content was greater in non-inoculated plants under either normal or drought conditions than in those treated with *R. arrhizus*. Therefore, the maximum carotenoid content was significantly observed under normal conditions (non-inoculated control) (0.33 ± 0.066 mg/g FW), whereas the lowest content was observed under the highest drought level in inoculated plants (0.21 ± 0.11 mg/g FW).

### **Influence of*R. arrhizus*on carbohydrate contents in*Zea mays*under drought stress**

The severe impact of drought stress was significantly reduced total carbohydrate content compared with that of the well-watered plants (non-inoculated control), as shown in Table 5. Notably, the endophytic colonization of *Zea mays* with *R. arrhizus* significantly increased total carbohydrate concentration in plants grown under both optimal irrigation conditions and drought conditions. This enhancement was not most pronounced under normal irrigation conditions, where inoculated plants exhibited higher carbohydrate levels (63.96 ± 0.69 and 57.66 ± 0.78 mg/g DW, respectively). Furthermore, carbohydrate content demonstrated an inverse relationship with the severity of drought stress, the lowest carbohydrate levels observed under the highest drought level (200 mL H_2_O irrigation). Therefore, the carbohydrate content decreased by 15.24% and 21% in inoculated and untreated plants under severe drought. Additionally, the total soluble sugar content in *Zea mays* subjected to drought stress significantly exceeded the insoluble sugar content in both plants treated with or without *R. arrhizus*. The highest concentration of soluble sugars (41.43 ± 0.74 mg/g DW) was observed in plant samples inoculated with *R. arrhizus* under mild drought stress, whereas the lowest concentration was found in non-inoculated plants under well-irrigated conditions (24.18 ± 0.78 mg/g DW). Furthermore, insoluble sugars were maximized in untreated plants under well-watered conditions and minimized under severe drought stress in non-inoculated plants (33.48 ± 0.65 and 8.64 ± 0.74 mg/g DW, respectively).

**Table 5:** Effect of inoculating *R. arrhizus* on total phenolic (mg GAE/g DW) and flavonoid contents (mg QE/g DW) of *Z. mays* plants under drought stress

Parameters Treatments	Total phenolic content (mg GAE/g DW)	Total flavonoid content (mg QE/g DW)
**T1**	16.950 ± 0.35f	0.052 ± 0.017e
**T2**	21.160 ± 0.42c	0.072 ± 0.025d
**T3**	21.350 ± 0.56b	0.238 ± 0.042b
**T4**	24.039 ± 0.25a	0.279 ± 0.036a
**T5**	18.653 ± 0.47e	0.072 ± 0.073d
**T6**	20.355 ± 0.52d	0.134 ± 0.034c

Data are the mean (± SE) for *n* = 3. One-Way ANOVA: Duncan multiple range test (DMRT) finds no significant difference (*p* < 0.05) between the means of the same column accompanied by the same letter. Treatments: T1-control (800 mL H₂O); T2– control with *Rhizopus*; T3– 400 mL H₂O; T4– 400 mL H₂O with *Rhizopus*; T5– 200 mL H₂O; T6– 200 mL H₂O with *Rhizopus*

### **Influence of*R. arrhizus*on total phenolic and total flavonoid contents in*Zea mays*under drought stress**

Drought stress, compared with the control, led to higher accumulation of total phenolic compounds and total flavonoids in maize plants, as shown in Table 6. Compared with non-biopriming, inoculation with the endophytic fungus *R. arrhizus* stimulated the biosynthesis of secondary metabolites (total phenolic compounds and total flavonoids) either under well-irrigated conditions or under drought stress. The maximum total phenolic and flavonoid contents were observed under moderate drought conditions (400 mL H_2_O) in inoculated plants (24.039 ± 0.25 mg GAE/g DW and 0.279 ± 0.036 mg QE/g DW, respectively) compared with those in non-inoculated plants under the same conditions (21.350 ± 0.56 mg GAE/g DW and 0.238 ± 0.042 mg QE/g DW, respectively). However, high drought stress reduced the total phenolic and flavonoid contents in inoculated plants to 20.355 ± 0.52 mg GAE/g DW and 0.134 ± 0.034 mg QE/g DW, respectively, whereas the total phenolic and flavonoid contents in non-inoculated plants were 18.653 ± 0.47 mg GAE/g DW and 0.072 ± 0.073 mg QE/g DW, respectively.

**Table 6:** List of the ISSR primer codes and sequences, as well as the number of monomorphic and polymorphic (unique and non-unique) bands produced by the ISSR analysis of the samples under study

No	Primes and number of their amplification DNA bands				Types of amplified bands						% of Polymorphism
	Primer code	Primer sequence	Amplicon lengths (bp)	% of amplified bands	Monomorphic bands	Polymorphic bands			Total no. of mono- and polymorphic bands	Polymorphic information content (PIC)	
						Unique band	Non-unique	Polymorphic bands			
**1**	MNLM-1	(TG)8AA	400–1000	19	3	1	0	1	4	0.078	25%
**2**	MNLM-2	(CTC)6	200–700	31	5	1	0	0	6	0.162	16.6%
**3**	MNLM-3	(TG)8AA	200–1500	33	4	1	1	2	6	0.144	33.3%
**4**	MNLM-4	(CA)8T	600–1000	17	2	1	0	1	3	0.102	33.3%
Total DNA bands				100	14	4	1	5	19	-	26.3%

### **Genomic DNA profiling by ISSR in*Zea mays*under drought stress**

**Fig. 6:**
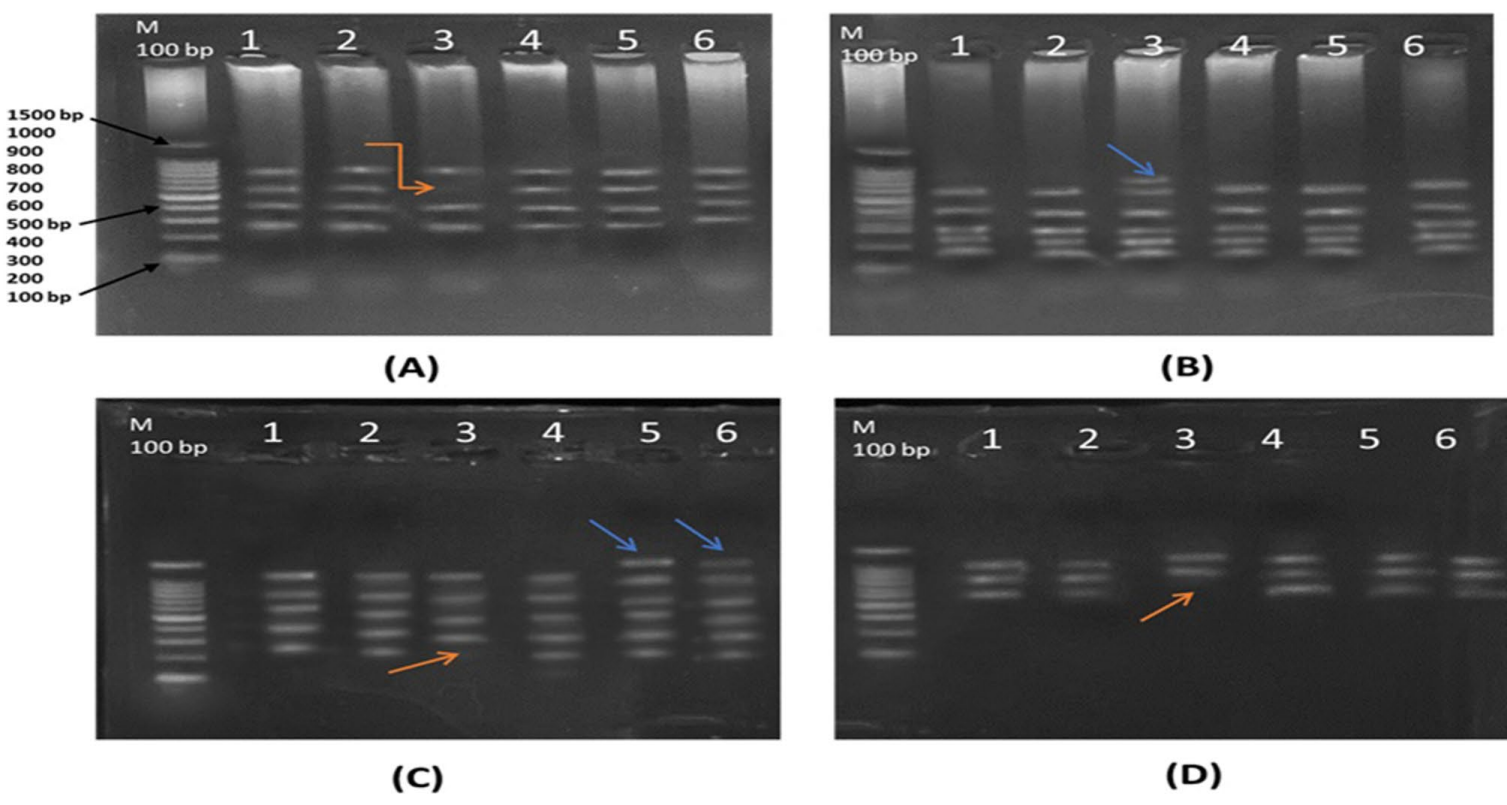
Products of genomic DNA using Inter Simple Sequence Repeats (ISSR) extracted from leaves of (*Z. mays*) at different treatments. The lanes referred to germplasm of 6 treatments. Lane M =1.5 Kb DNA marker. Treatments: **T1**-control (800 mL H₂O); **T2**– control with *Rhizopus*; **T3**– 400 mL H₂O; **T4**– 400 mL H₂O with *Rhizopus*; **T5**– 200 mL H₂O; **T6**– 200 mL H₂O with *Rhizopus*

**Fig. 7:**
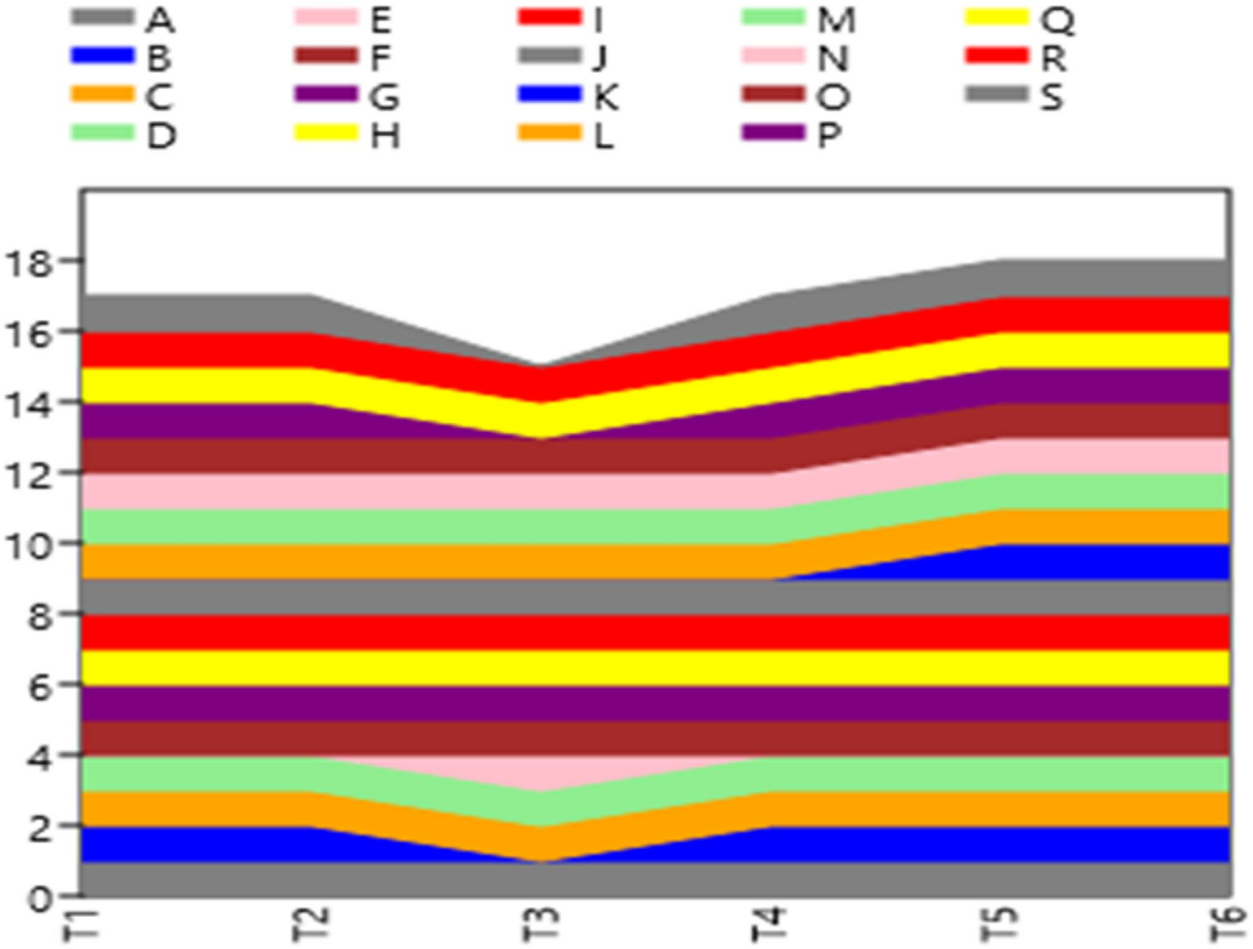
A stacked area created with PAST-pc that displays the band number of amplified DNA markers produced by four ISSR primers in the samples under study.Treatments: **T1**-control (800 mL H₂O); **T2**– control with *Rhizopus*; **T3**– 400 mL H₂O; **T4**– 400 mL H₂O with *Rhizopus*; **T5**– 200 mL H₂O; **T6**– 200 mL H₂O with *Rhizopus*

The inter simple sequence repeat (ISSR) technique was used to analyze the impact of drought stress on the genetic material of *Zea mays* plants compared with those grown under well-watered conditions. A total of eight primers were initially used to assess genetic polymorphisms in the fresh leaves of *Zea mays*, as summarized in Table 7. However, only four primers produced consistent and reproducible bands, which are depicted in Figs. 6 and 7. The specific codes and sequences of the ISSR primers used in the present study are also detailed in Table 7.

**Table 7:** Band sharing index (BSI), number of new bands that appeared (**a)**, and number of bands that disappeared (**b**), as related to the control and genome template stability (GTS) percentage in *Zea mays* L. seedlings after treatments by ISSR marker

Primers	No.of bands in control	Treatments									
		Control + Rhizopus		400 mL H_2_O		400 mL H_2_O + Rhizopus		200 mL H_2_O		200 mL H_2_O + Rhizopus	
		a	b	a	b	a	b	a	b	a	b
**MNLM-1**	4	0	0	0	1	0	0	0	1	0	0
**MNLM-2**	5	0	1	0	0	0	1	0	1	0	1
**MNLM-3**	5	0	0	0	1	0	0	0	1	0	1
**MNLM-4**	3	0	0	0	1	0	0	0	1	0	0
**Total**	17	0	1	0	3	0	1	0	4	0	2
**a + b**	-	1		3		1		4		2	
**GTS%**	100%	94.11%		82.35%		94.11%		76.47%		88.23%	

Altogether, the use of these four primers yielded 100 amplified DNA bands, which were subsequently analyzed for genetic variability. A total of 19 mono- and polymorphic DNA bands were identified, with a relatively low overall polymorphism value of 26.3%. Among the primers, MNLM-3 and MNLM-4 generated the highest polymorphism values, recorded 33.3%, whereas MNLM-2 presented the lowest polymorphism value of 16.6%. The observed polymorphisms were characterized by specific random DNA sequences, varying types of DNA bands (including unique, nonunique, and monomorphic bands), and differences in band intensity and length, which ranged between 0.2 and 0.9 Kbp, as outlined in Table 8. These findings highlight the ability of the ISSR technique to detect genetic variability in *Z. mays* under drought stress conditions.

**Table 8:** The Kulczyuski index was used to determine the similarity index for the ISSR primer code in the samples under study. The results are shown as the similarity index percentage

Samples	T1	T2	T3	T4	T5	T6
**T1**	1					
**T2**	1	1				
**T3**	0.88	0.88	1			
**T4**	1	1	1	1		
**T5**	0.97	0.97	0.8	0.97	1	
**T6**	0.97	0.97	0.84	0.97	1	1

**Fig. 8:**
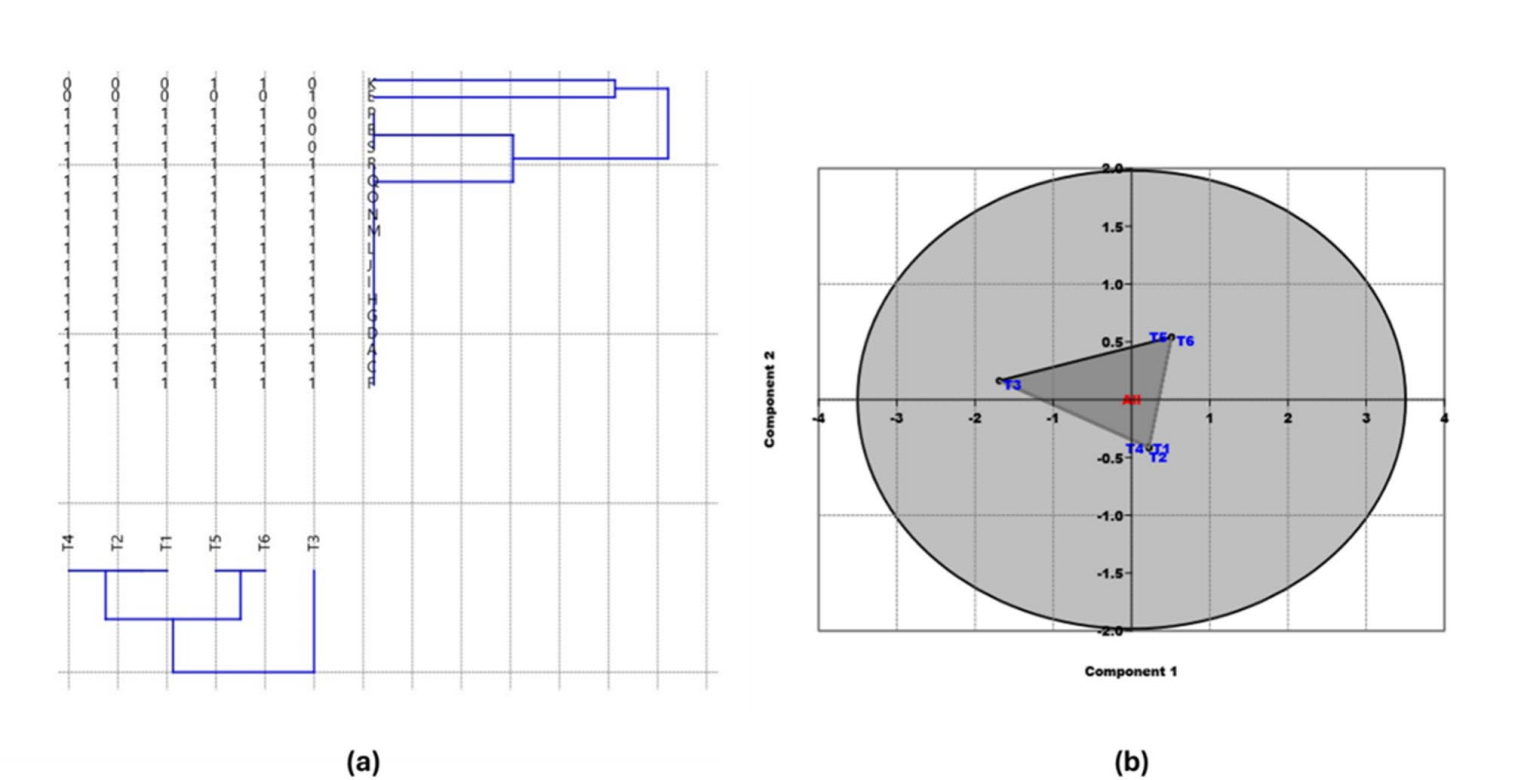
**(a)** Euclidean-based UPGAMA distance tree built using PAST-pc that displays the genetic separation among various treatments **(b)** PCA built with PAST-pc that displays the band number of amplified DNA markers produced by the ISSR marker.Treatments: **T1**-control (800 mL H₂O); **T2**– control with *Rhizopus*; **T3**– 400 mL H₂O; **T4**– 400 mL H₂O with *Rhizopus*; **T5**– 200 mL H₂O; **T6**– 200 mL H₂O with *Rhizopus*

Clustering analysis performed via PAST software and the Euclidean equation resulted in the formation of two major database-based clusters, labeled I and II (Fig. 8a). Cluster I was further separated into two distinct sub-clusters, which included treatments T1, T2, and T4. In contrast, cluster II included treatments T5 and T6, which were grouped together. Additionally, a third cluster (III), comprising only treatment T3, was identified. Principal component analysis (PCA) illustrated the relationships between the treatments on the basis of ISSR fingerprinting polymorphisms (Fig. 8b). The PCA scatter plot separated the treatments into three subgroups, which aligned precisely with their grouping in the clustering analysis.

The molecular findings in Table 8 indicate that the percentage of genetic template stability (GTS) was greatest in the groups treated with *Rhizopus*. The highest GTS percentage was observed in the control + *Rhizopus* treatment and the 400 mL H_2_O + *Rhizopus* treatment, both at 94.11%, followed by the 200 mL H_2_O + *Rhizopus* treatment at 88.32%. The lowest percentage of GTS was observed in the most severe drought treatment (200 mL H_2_O), at 76.47%, followed by the mild drought stress treatment (400 mL H_2_O), at 82.35%. Compared with that under drought stress alone, the proportion of stable genetic template application was greater in all the *Zea mays* with *Rhizopus* treatments under drought stress. This outcome was particularly noteworthy, as *Rhizopus* yielded stable genomic DNA, whereas drought stress diminished DNA stability.

Genetic similarities among the treatments were calculated via the Kulczyuski-similarity index. The highest genetic similarity observed was 0.97, whereas the lowest genetic similarity recorded between treatments T3 and T5 was 0.80. These results are summarized in Table 9. This analysis highlights the genetic variation among the treatments and confirms the consistency between clustering analysis and PCA in grouping the treatments on the basis of their ISSR polymorphism.

**Table 9:** Two-way ANOVA analysis evaluated the impact of *R. arrhizus* inoculation, drought stress conditions, and their interactions on several growth, metabolic parameters, and stress markers in *Z. mays* plants

*p*-value			
Parameters	*R*. arrhizus	Drought	*R*. arrhizus * Drought
**Shoot length**	0.000^*^	0.174^ns^	0.107^ns^
**Root length**	0.000^*^	0.112^ns^	0.021^*^
**Leaf length**	0.000^*^	0.430^ns^	0.513^ns^
**Shoot FW**	0.000^*^	0.000^*^	0.000^*^
**Root FW**	0.000^*^	0.001^*^	0.000^*^
**Shoot DW**	0.000^*^	0.009^*^	0.032^*^
**Root DW**	0.000^*^	0.000^*^	0.002^*^
**CAT**	0.000^*^	0.000^*^	0.003^*^
**POX**	0.010^*^	0.000^*^	0.725^ns^
**PPO**	0.009^*^	0.000^*^	0.500^ns^
**Proline**	0.000^*^	0.000^*^	0.046^*^
**Protein**	0.000^*^	0.000^*^	0.100^ns^
**H**_**2**_ **O**_**2**_	0.000^*^	0.005^*^	0.000^*^
**MDA**	0.000^*^	0.000^*^	0.000^*^
**Chl a**	0.000^*^	0.000^*^	0.000^*^
**Chl b**	0.000^*^	0.000^*^	0.000^*^
**Carotenoids**	0.000^*^	0.000^*^	0.004^*^
**Souble sugars**	0.000^*^	0.000^*^	0.000^*^
**Insouble sugars**	0.061^ns^	0.000^*^	0.000^*^
**Total carbohydrates**	0.000^*^	0.000^*^	0.135^ns^
**TFC**	0.162^ns^	0.000^*^	0.826^ns^
**TPC**	0.000^*^	0.000^*^	0.350^ns^

* Significant at the *p* < 0.05; ns non-significant

### **Statistical analysis**

**Fig. 9:**
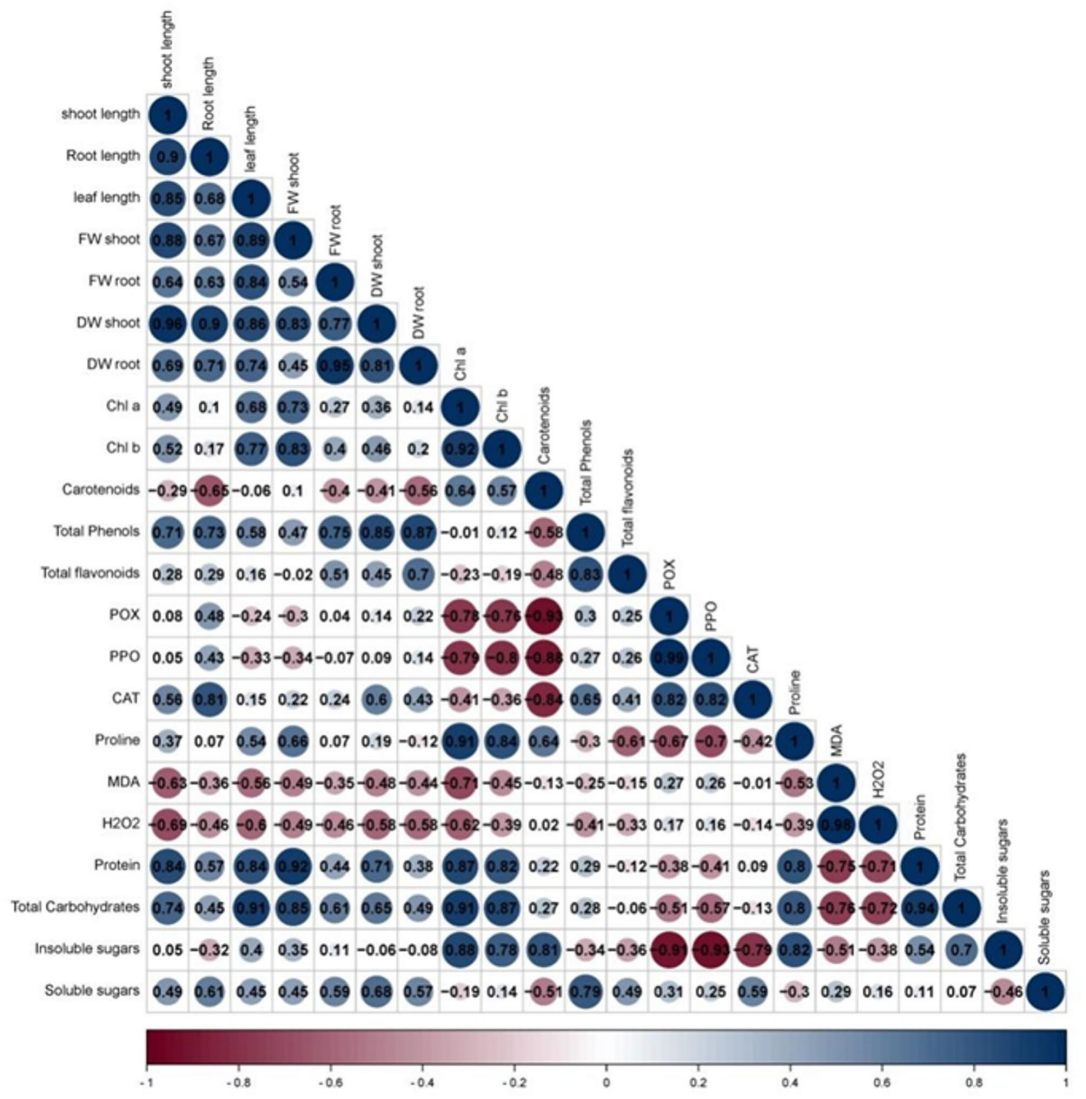
Pearson correlation among morphological and biochemical parameters for *Z. mays* plant after treatments. The boxed points correspond with significant correlation at *p* < 0.05 (blue: positive; red: negative)

The Pearson correlation coefficient for color between the morpho-biochemical factors analysis was examined and is depicted in Fig. 9. The strongest positive correlation for the morphological data was 0.96 between the shoot length and dry weight of the shoot, followed by 0.95 between the fresh weight of the root and dry weight of the root. The strongest positive correlation in the biochemical data was 0.91, which was observed between proline and chlorophyll a, as well as between total carbohydrates and chlorophyll a. The total phenols and flavonoids examination revealed a peak positive correlation of 0.93 between the POX enzyme and carotenoids, as well as between the PPO enzyme and insoluble sugars. The lowest positive significant correlation coefficient was 0.07, which was observed between root length and proline content, as well as between proline content and the fresh weight of the roots. Conversely, the lowest negative correlation was − 0.01, which was noted between malondialdehyde and catalase enzyme.

**Fig. 10:**
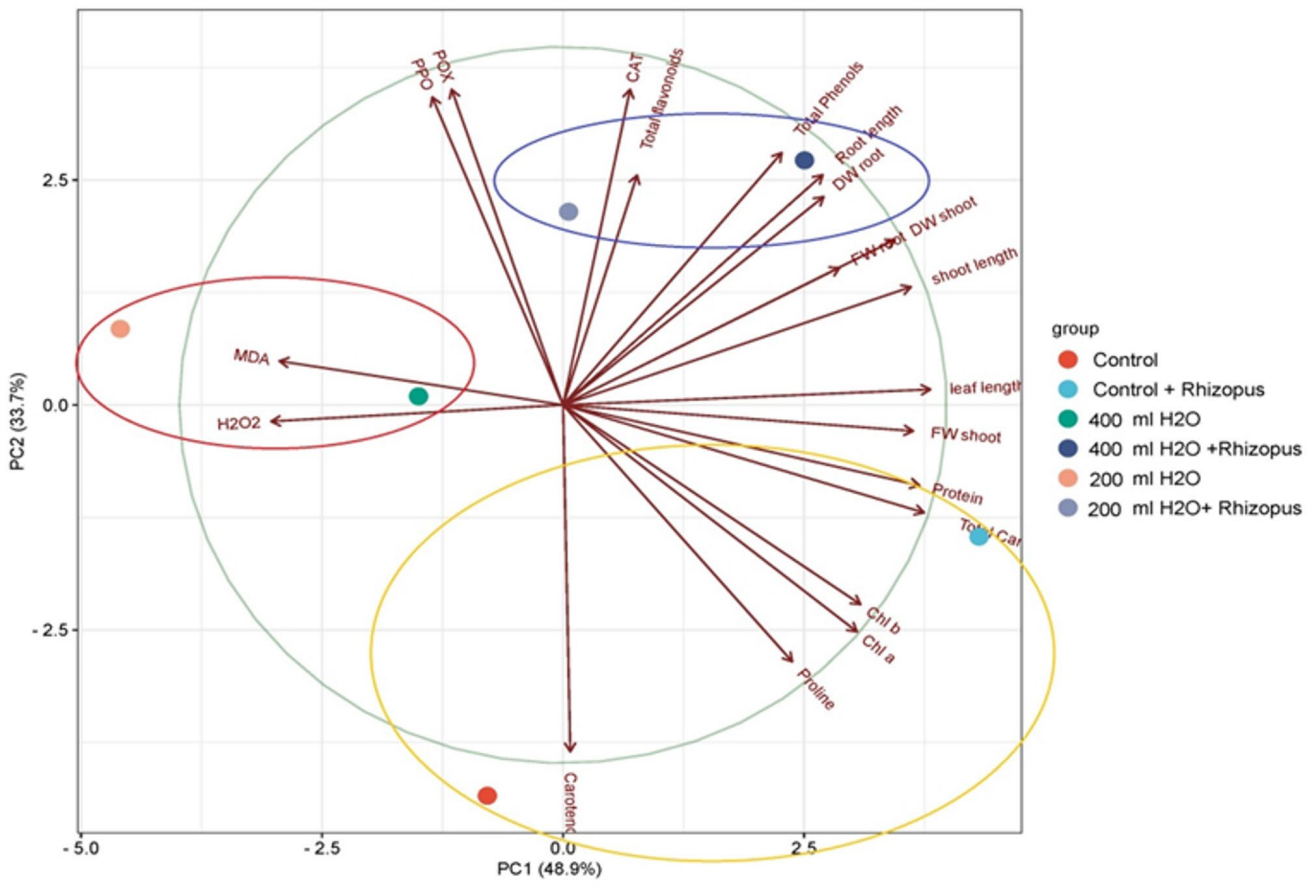
Principal component analysis (PCA) among different treatments of *Z. mays* based on morphological and biochemical analyses

Principal component analysis (PCA) is depicted in Fig. 10, which shows the ordination of the various treatments of *Zea mays* on the basis of morpho-biochemical investigations. PCA1 accounted for 48.9%, whereas PCA2 represented 33.7%. The analyzed treatments were categorized into three groups. The first group, situated in the positive right quadrant of the PCA, comprised *Zea mays* subjected to drought stress and *Rhizopus* (200 mL H_2_O + *Rhizopus* & 400 mL H_2_O + *Rhizopus*), characterized by CAT enzyme activity and total phenolic content. The second group, positioned in the negative left quadrant of the PCA, included *Zea mays* treated solely with drought stress (200 mL H_2_O & 400 mL H_2_O), distinguished by the MDA and H_2_O_2_ levels. The third group, situated in the lower quadrant of the PCA, comprised the control and control + *Rhizopus* groups and was characterized by the presence of carotenoids, proline, chlorophyll a, and chlorophyll b.

## Discussion

The symbiotic relationship between plants and endophytic fungi has gained considerable attention in recent years because of its potential applications in promoting environmentally friendly agriculture, especially in mitigating the impacts of climate change on drought stress [42]. The successful colonization of maize roots by *Rhizopus* highlights their active association and functional symbiosis. Microbiological colonization patterns and effectiveness are highly specific and variable, ranging from mutualistic to neutral or pathogenic, depending on the plant and microbial species involved [27]. Drought stress in plants causes oxidative stress through the excess production of reactive oxygen species (ROS), leading to structural and functional damage. ROS compromise membrane integrity, enzyme activity, and the cell cycle, often causing mutagenesis and cell death, ultimately affecting plant survival [43].

All the morphological parameters in the current study, including shoot, root, and leaf lengths, as well as fresh and dry weight of shoot and root, showed an observable decrease under severe drought stress (200 mL H₂O). These results corroborate Abobatta’s [44] finding that drought stress impairs cell expansion and growth due to reduced turgor pressure, while treatment with *R. arrhizus* boosted these growth indices. Endophytes possess numerous beneficial impacts, including promoted plant growth, improved nutrient uptake, and increased abiotic stress tolerance [45]. Similarly, Shaik and Thomas [46] found that inoculating tomato seeds with endophytes increased the dry weight of both the roots and leaves. Root growth promotes nutrient absorption, resulting in higher overall plant biomass [47]. Moreover, arbuscular mycorrhizal (AM) fungi have been demonstrated to increase root biomass during drought conditions [48].

In the present study, *Zea mays* treated with *R. arrhizus* under moderate and severe drought stress exhibited a notable increase in the activity of antioxidative enzymes (CAT, POX, and PPO); this modulation is essential for increasing plant resistance to abiotic stress [49, 50]. Drought stress frequently induces oxidative stress through the over-accumulation of ROS, exceeding the plant’s antioxidant defense capabilities. Antioxidant enzymes are essential for alleviating oxidative damage. CAT is vital in directly converting hydrogen peroxide (H₂O₂) into water and oxygen. Its function is crucial for ROS detoxification under drought conditions and may safeguard chloroplasts (primary sites of ROS production) during stress [51]. POX, an enzyme containing heme, facilitates the oxidation of organic substrates in the presence of H₂O₂. Elevated POX activity is frequently correlated with ROS accumulation [52]. PPO facilitates the hydroxylation of monophenols and the oxidation of o-diphenols to o-quinones. PPO is associated with tissue browning and also plays a role in drought tolerance [53]. Its exact role under drought remains unclear, but evidence suggests involvement in defense mechanisms. Similar results have been observed in *Malva parviflora* bioprimed with *Beauveria bassiana* under drought conditions [54]. Furthermore, research has demonstrated that *S. indica*-inoculated tomato plants also exhibit increased antioxidative activity under drought conditions [55].

In the present study, compared with nontreated plants, *Zea mays* bioprimed with *R. arrhizus* presented increased proline and protein contents under severe drought stress. Proline scavenges ROS, including H_2_O_2_ and O_2_¯, produced during drought stress, thereby relieving oxidative stress and protecting the plant from associated damage [56]. Similar findings have been reported for maize inoculated with *P. indica*, where increased proline accumulation contributes to improved drought tolerance [57]. Proline performs multiple roles in drought responses, acting as an osmolyte, scavenging free radicals, stabilizing cellular membranes, and regulating redox potential within cells [58]. Several studies have highlighted the importance of microbial activity in the rhizosphere in influencing antioxidant responses to drought conditions by modulating proline and protein levels in plants [59]. Solutes such as proline reduce the cellular water potential by interacting with water molecules, which helps maintain protein and membrane integrity, thereby minimizing cellular damage under drought conditions [60, 61]. In general, the total phenol content, soluble sugar content, and proline content of plants injected with endophytes significantly increase when subjected to severe stress, such as drought [23].

Malondialdehyde (MDA) content is an important marker for assessing plant responses to drought stress; elevated H₂O₂ levels trigger lipid peroxidation and membrane damage, serving as cellular markers for oxidative stress [62]. Studies have consistently indicated that drought stress increases MDA concentrations [63, 64]. However, the current results revealed that while drought stress increased the MDA and H_2_O_2_ contents, inoculation with *R. arrhizus* significantly reduced the MDA content under drought stress. The reduction in MDA content may be due to the increment in the production of antioxidant enzymes (CAT, POX, and PPO) which act as ROS scavengers and reduce lipid peroxidation. Similar results have been shown in rice plants, where *Trichoderma* species treatment reduced H₂O₂ and MDA contents under drought stress [65]. Similarly, *P. indica* colonization of *Oryza sativa* plants resulted in lowered MDA levels under both well-watered and water-stressed conditions [66]. These results underscore the potential of endophytic fungi such as *R. arrhizus* to minimize oxidative stress and increase drought resistance in plants. Additionally, Kaboosi et al. [67]. reported that inoculation with the endophyte *S. indica* decreased the MDA content in rice plants under drought conditions.

Several studies have documented a decrease in chlorophyll levels under drought stress [68, 69]. This negative impact may be attributed to the inhibitory effect on chlorophyll biosynthesis enzymes, the stimulatory effect of chlorophyllase, and the massive generation of ROS [70]. Additionally, drought stress suppresses carbon assimilation in plants and harms the photosynthetic apparatus [71]. Multiple findings have highlighted the positive impact of endophytic fungal inoculation on chlorophyll fluorescence and chlorophyll content under drought conditions [72, 73]. Halo et al. [74]. reported that the inoculation of tomato plants with *Talaromyces omanensis* increased the chlorophyll a and chlorophyll b contents and carotenoid content under both normal and drought conditions. Similarly, compared with drought stress alone, the fungal combination of paddy plants and drought conditions maximized the chlorophyll a and chlorophyll b concentrations. Additionally, the combination of sunflower with *Fusarium proliferatum* increased the chlorophyll a and chlorophyll b contents under drought conditions [75]. On the other hand, drought stress had no significant effect on the contents of chlorophyll a and chlorophyll b in *Chenopodium quinoa*, as their levels remained unchanged under both drought and non-drought conditions [76]. Moreover, minimal changes in the total chlorophyll content of banana plants were detected in both inoculated and non-inoculated plants, and a 50% decrease was detected in maize plants inoculated with endophytic fungi [77]. Several studies have reported that arbuscular mycorrhizal (AM) symbiosis under drought conditions leads to increased carotenoid concentrations in host plants [78, 79], indicating improved drought tolerance [80]. However, in the present study, carotenoid content was higher in non-inoculated plants under both normal and drought conditions compared to those inoculated with *Rhizopus* sp. A similar finding was obtained by [81] indicating that the concentration of carotenoids was elevated in the roots and leaves of drought-stressed plants compared to control and typical arbuscular mycorrhizal (AM) plants. Such increment was attributed to the enhanced levels of ROS, specifically hydrogen peroxide, which were found to be higher in drought conditions than in the control and AM plants. Furthermore, the singlet oxygen species have been associated with the augmented activity of carotenoids and the conversion of CO_2_ into starch, as documented by Ramel et al. [82]. Horvath et al. [83]. obtained similar results by studying how mycorrhizal inoculations affect carotenoid synthesis in field-grown tomatoes. Genes implicated in carotenoid production are increased in response to drought and thermal stress. In sweet pepper, the expression of violaxanthin deepoxidase and lycopene epsilon cyclase is markedly enhanced under salt and drought stress, resulting in elevated carotenoid levels and improved stress adaptation [84]. Likewise, Shao et al. [85]. demonstrated overexpression of carotenoid production and promoted tolerance to abiotic stress due to enhancement of IbPSY1.

The reduction in carbohydrate content with drought stress may be attributed to the damage to macromolecules, such as carbohydrates, caused by the accumulation of ROS under severe drought conditions [86]. Similarly, the biopriming of *Malva parviflora* with *Beauveria bassiana* increased the accumulation of carbohydrates in the leaves of the plants under drought conditions compared with the nontreated plants. The accumulation of total carbohydrates in the plants inoculated with endophytic fungi assisted the plants in effectively tolerating drought stress more than did the inoculation-free plants [54]. Symbiotic fungi improve plant drought tolerance by reducing water consumption in inoculated plants compared with non-inoculated plants [87, 88]. Additionally, the colonization of plants with endophytic fungi improves plant drought tolerance through the production of certain solutes, such as sugars that balance the plant osmotic capacity [25, 89, 90]. In addition, the antioxidant activity of some endophytic fungi stimulates their plant host’s resistance against drought stress through the production of sugars, phenols, and carbohydrates [91, 92]. *Moringa oleifera* with *Aspergillus aculeatus* (TL3), *Meyerozyma guilliermondii* (TG), and *Microdochium majus* (WA) isolates increased the synthesis of total soluble sugars irrespective of polyethylene glycol treatment. The ability of drought to reduce soluble sugars may be due to the overproduction of abscisic acid, which results in prolonged closure of plant stomata, the inhibition of photosynthesis, a decrease in soluble sugar synthesis, and a reduction in plant biomass. The accumulation of soluble sugars in inoculated plants, in contrast to non-inoculated plants, may be attributed to the production of a sufficient quantity of abscisic acid, which moderates stomatal closure and hence activates photosynthesis, stimulates the synthesis of soluble sugars, and enhances biomass production [19].

Plant inoculation under stressful conditions with endophytic fungi was reported by Verma et al. [87] to reduce the adverse effects of unfavorable conditions by regulating the production of secondary metabolites such as phenolic compounds. The present study revealed that the inoculation of *Zea mays* with *R. arrhizus* stimulated the biosynthesis of secondary metabolites, including total phenolic and flavonoid compounds. Similarly, Gul et al. [93]. reported that *Aspergillus welwitschiae* BK isolate increased the total phenolic and flavonoid contents in maize plants under both control and salt stress conditions compared with those in non-inoculated plants. The proliferative effect of endophytic fungal inoculation on the total phenolic and flavonoid contents of plants under stress conditions has been proven in previous studies [30, 94, 95]. Inoculation of plants with endophytic fungi during stress conditions regulates the production of potent antioxidant phenolic compounds that play essential roles in mitigating membrane damage and scavenging harmful ROS [96–98].

Inter simple sequence repeat (ISSR) DNA analysis has been demonstrated to be an effective approach for confirming genetic homogeneity in plants subjected to various heavy metals and abiotic stresses [32, 99–102]. This technique is particularly valuable for assessing polymorphisms and genetic similarity among individuals, even when they exhibit phenotypic differences. Importantly, the highest degree of genetic similarity observed in such treatments indicates that, while individuals may appear different phenotypically, they are genetically very similar. These phenotypic differences are often attributed to environmental influences, which are not accounted for when determining genetic similarity [33]. The emergence and disappearance of specific DNA bands in ISSR profiles can provide insights into the effects of stress on genetic material. As noted by Labra et al. and Al-Qurainy [103, 104], ISSR analysis has shown that exposure to stress, particularly at high concentrations, induces changes in DNA in various target sequences, which can result in genotoxic effects. These changes are reflected in the formation of new DNA bands in the ISSR profile, as well as the lack of certain typical bands. These phenomena are often interpreted as mutations, likely caused by genetic variations arising from DNA rearrangements or damage [105].

The current study showed that only 4 out of the 8 ISSR primers produced clear and reproducible amplification patterns. Several factors may have contributed to this outcome, including the possibility of suboptimal DNA quality, primer-template mismatch, or the intrinsic specificity of the primers used. Additionally, drought-induced alterations in DNA conformation or chromatin structure may have affected primer binding efficiency. These limitations highlight the sensitivity of ISSR markers to both technical and biological variables [106].

The use of ISSR primers has revealed notable differences in the extent of genetic variation among genotypes. These variations are influenced by the genetic makeup of individuals and the environmental conditions to which they are subjected. Furthermore, it is expected that genetic variety within populations may decline over time because of these stress-induced genetic alterations and the loss of certain genetic traits [107]. This highlights the potential of the ISSR as an effective instrument for understanding the impact of abiotic stressors as well as for monitoring genetic stability and diversity under varying environmental conditions.

The polymorphism percentage obtained in this study (26.3%) is relatively low compared to some previous ISSR-based studies on maize. For example, Soliman et al. [108]. reported a polymorphism rate of 89.59% using ISSR. However, it is important to note that other studies have also reported lower polymorphism rates. In a study by El-Akkad et al. [109]., a 36.46% polymorphism was found in maize, which is similar to our findings. These variations in polymorphism levels can be attributed to factors such as the number of primers used, plant genotype, and the intensity of the stress applied. Despite the relatively low polymorphism percentage in our study, the specific appearance and disappearance of bands suggest significant genomic changes. Thus, even moderate levels of polymorphism can indicate stress-induced genomic instability and support our conclusions.

Alterations at the DNA level, such as damage and instability, can significantly influence the integrity of the genomic template. The evaluation of genotoxic effects allows for the determination of qualitative changes in genomic template stability (GTS) [110, 111]. The genomic template stability assay serves as a qualitative method for detecting changes in ISSR profiles caused by *Rhizopus* and varying levels of drought stress relative to the profiles obtained from the control sample. Genomic template stability (GTS) can be influenced by DNA damage, as shown in previous studies [112, 113]. This investigation revealed that the stability of the genetic template varied across the different treatments. The lowest value observed was 76.74% in non-inoculated samples under severe drought conditions, whereas the highest value of 94.11% was noted in inoculated samples under well-watered and mild drought conditions.

The application of ISSR fingerprinting in this study proved essential for detecting genetic variability and stress-induced genomic responses influenced by endophytic fungi. The current results demonstrate a direct genomic-level alteration associated with fungal colonization. The observed genetic polymorphism and clustering patterns highlight the role of endophytes in modulating plant genome activity under drought, offering novel insights into plant-microbe interaction dynamics. This provides a new layer of understanding that complements existing literature and supports the potential of ISSR as a rapid and cost-effective tool for evaluating endophyte-mediated stress tolerance [114, 115].

The morpho-biochemical studies of *Zea mays* under various treatments exemplify a field where multivariate analysis techniques, including principal component analysis (PCA) and Pearson color correlation, are widely utilized. These methods are employed for the identification, classification, and modeling of data [116]. Principal component analysis (PCA) is a statistical technique that proficiently identifies the key principal components responsible for the majority of information within a dataset, thereby reducing the number of features present in the dataset [117, 118]. The present investigation classified the studied *Zea mays* under different treatments into three groups on the basis of the different morphological and biochemical parameters of the maize.

The distinct separation of T3 in both the clustering and PCA analyses indicates a marked physiological deviation compared to the other treatments. This divergence can be attributed to the fact that T3 was subjected to drought stress without any microbial inoculation, potentially representing a threshold level of abiotic stress beyond which the plant’s physiological responses were significantly altered. The absence of beneficial microbial support under drought conditions likely exacerbated the stress effects, leading to impaired physiological functions and a unique metabolic profile. This highlights the critical role of microbial inoculation in mitigating drought-induced damage and maintaining physiological stability under stress conditions.

The colonization of *R. arrhizus* is strongly associated with increased morphological parameters (shoot/root length, biomass (fresh and dry), and leaf length) in water-stressed plants (T4 and T6), most likely due to improved water intake and root structure. The fungus also showed a positive correlation with chlorophyll and carbohydrate levels, implying improved photosynthesis and energy maintenance. Increased soluble sugars under inoculation act as osmoprotectants against drought. In contrast, drought stress alone (T3, T5) revealed negative relationships with protective antioxidants (phenolics and flavonoids), which were dramatically boosted by *R. arrhizus* (particularly in T4), demonstrating its function in mitigating oxidative damage. ISSR molecular markers demonstrated high genetic polymorphism and similarity indices among inoculation treatments (T2, T4, T6), indicating that *R. arrhizus* exhibits a systemic effect in inducing genetic and physiological stability during drought. Overall, *R. arrhizus* appears to improve drought tolerance via a variety of mechanisms, including growth promotion, physiological balance maintenance, osmoprotection, and oxidative damage reduction, which is consistent with previous findings [57, 65, 74].

## Materials and methods

### **Isolation of endophytic fungi and deposition into GenBank**

In autumn 2024, thirty-day-old *Zea mays* plants were sampled from a research area within the Faculty of Science, Zagazig University, El-Sharkia Governorate, Egypt (30.5872°N, 31.5036°E). Three plant samples were collected and immediately processed for the isolation of endophytic fungi. Following the method outlined by Ismaiel et al. [119]., *Zea mays* fully flowering plants were subjected to a surface sterilization procedure by cleaning under running water to remove dust and debris. Followed by air-drying and submersion in 70% ethanol for 1 min. The samples were then immersed in a 5% sodium hypochlorite solution for 1 min, drained thoroughly, and rinsed three times with 70% ethanol for 30 s. Following sterilization, the plant material was carefully cut into small pieces (2–3 cm) under sterile conditions and washed every 4-6 intervals with sterilized distilled water. After the final rinse, the pieces were gently wiped onto sterile filter paper [120]. The sterilized plant pieces were transferred to Petri dishes containing potato dextrose agar (PDA) medium, which was composed of 200 g/L potato, 20 g/L dextrose, and 20 g/L agar with a pH of 6.0. The PDA was supplemented with the streptomycin sulfate (250 µg/mL, Sigma) to prevent bacterial contamination. Plates were incubated at 28 ± 2 °C for 7 days. After this, hyphae development was observed, and the resulting cultures were subcultured onto fresh PDA plates for purification. Finally, the selected fungal isolate was preserved as plate and slant cultures at 4 °C and subsequently identified morphologically and molecularly.

For molecular identification, the fungal isolate was subcultured onto PDA media and incubated at 25 °C for 7 days. Genomic DNA was extracted using the Cetyltrimethylammonium bromide (CTAB) method, which involves breaking down mycelial cell walls with liquid nitrogen [121]; then the mixture was incubated at 65 °C. The extracted DNA was then dispersed in 50 µL of sterilized distilled water. To confirm its identity, the DNA was amplified via the ITS1 (5′-TCCGTAGGTGAACCTGCGG-3′) and ITS4 (5′-TCCTCCGCTTATTGATATGC-3′) primers [122], following the protocol established by White et al. [123]. The phylogenetic analysis was conducted using MEGA software (Molecular Evolutionary Genetics Analysis). Sequences were aligned and analyzed in MEGA to construct the phylogenetic tree using Neighbor-Joining (NJ). The sequences used in the analysis were retrieved from the NCBI GenBank database, which is accessible through the following link: https://www.ncbi.nlm.nih.gov/nuccore/PQ882778.

### **Plant material and growing conditions**

The experiment was conducted at the Faculty of Science, Botany and Microbiology Department, Zagazig University. High-quality maize seeds (*Zea mays* L.) var. Giza 2 were obtained from the Crop Institute, ARC, Giza, Egypt, with a high genetic and physical purity and low inert matter content. The seeds were subjected to a five-min surface sterilization with 1.5% sodium hypochlorite (NaOCl) solution and washed at respective times with sterilized distilled water prior to sowing. The maize seeds were bioprimed by immersing them in a freshly made suspension of *R. arrhizus* spores (about 10⁸ spores/mL) for 12 h before seeding. When the seedlings reached the four-leaf stage, two weeks after the sterilized seeds were sown, drought stress was induced. Drought stress was imposed by regulating irrigation volumes: control plants received full irrigation (800 mL per pot), while stress treatments were subjected to moderate (400 mL) and severe (200 mL) water levels, applied at consistent intervals to simulate field-relevant drought conditions. Each treatment was conducted in plastic pots (25 cm diam.), each filled with 2.5 kg of sterilized homogenized loamy soil. The experiment comprised six treatments, each replicated three times biologically (Table 1). After 40 days, the plants were harvested to evaluate morphological and biochemical-molecular indices.

### **Colonization of*Zea mays*roots by*Rhizopus arrhizus***

Fresh *Zea mays* roots were rinsed under running tap water for 5 min, followed by surface sterilization by immersion in 70% ethanol (10 s), and thoroughly washed with sterile distilled water (5 min). To assess fungal colonization efficacy, root segments (1–2 cm in length) were aseptically transferred to PDA plates. The cultures were incubated at 28 °C ± 2 °C for 7 days, after which fungal growth was analyzed.

### **Determination of various plant growth parameters**

#### **Measurement of morphological parameters**

*Zea mays* plants were harvested from all treatments, and then distilled water was used to rinse the roots of all plants in order to remove any soil particles. Fresh weights (FW) of shoot and root parts were measured immediately. The lengths of shoots, roots, and leaves were measured. For future biochemical analysis, fresh plant specimens were frozen at temperatures ranging from − 10 °C to -25 °C. Samples were subjected to dryness in an oven for 72 h at 65 °C for the determination of dry weights (DW).

### **Measurement of the biochemical traits of*Zea mays*plants under drought stress conditions**

#### **Proline content**

The proline content was determined following the methods of Bates et al. [124]., with slight modifications. The proline content of fresh leaves was extracted using a 3% (w/v) sulphosalicylic acid solution, and the resulting mixture was filtered. Two mL of glacial acetic acid and 2 mL of acid ninhydrin were added to 2 mL of the filtered extract. The final mixture was heated in boiling water for 1 h and subsequently cooled in an ice bath. After adding 4 mL of toluene and vortexing for 20–30 s, the absorbance of the upper layer was measured at 520 nm.

### **Hydrogen peroxide quantification**

Hydrogen peroxide (H₂O₂) levels were measured via a protocol adapted slightly from Velikova et al. [125]. H₂O₂ levels in fresh leaves were determined by grinding samples in 5 mL trichloroacetic acid (TCA) and then centrifuged at 8000 rpm for 15 min. The supernatant (0.5 mL) was then mixed with 0.5 mL of potassium phosphate buffer (pH 6.8, 10 mM) and 1 mL of potassium iodide (1 M). The amount of H₂O₂ was quantified by measuring the absorbance at 390 nm. H_2_O_2_ (mg/g FW) content was calculated by a standard curve created with known H₂O₂ concentrations (1, 5, and 10 mM H_2_O_2_).

### **Malondialdehyde assay (lipid peroxidation)**

The content of malondialdehyde (MDA), a lipid peroxidation biomarker, was determined following the methods of Heath and Packer [126]. Leaf tissues were blended with 2 mL of 20% trichloroacetic acid (TCA) (w/v) containing 1% thiobarbituric acid (TBA) (w/v) and then heated at 95 °C for 30 min. The absorbance of this mixture was measured at 532 nm, and a non-specific reading at 600 nm was subtracted. The MDA concentration was then calculated using its molar extinction coefficient (155 mM⁻¹ cm⁻¹) and expressed as nM/g FW.

### **Extraction and assay of antioxidant enzyme activities**

Fresh leaves (1 g) from *Zea mays* were homogenized in 10 mL of 50 mM phosphate buffer (pH 7.0) containing 1% (w/v) polyvinylpyrrolidone and 0.1 mM ethylenediaminetetraacetic acid (EDTA). The resulting extracts were centrifuged at 4 °C for 10 min at 8,000 rpm to obtain the supernatant, which was then used to assess antioxidant enzyme activities. Catalase (CAT) activity was determined according to Aebi [127] by measuring the decrement in absorbance at 240 nm resulting from H_2_O_2_ decomposition. The reaction mixture consisted of 1.9 mL of 50 mM potassium phosphate buffer (pH 7.0), 100 µL of enzyme extract, and 1 mL of 0.3% hydrogen peroxide (H₂O₂).

Peroxidase (POX) activity was measured, as described by Maehly [128]. POX activity was quantified by observing the increase in light absorption at 470 nm. The reaction solution contained 50 mM phosphate buffer (pH of 7.0), 1 mM guaiacol, and 0.5 mM hydrogen peroxide (H₂O₂).

Polyphenol oxidase (PPO) activity was assayed using the methods of Kar and Mishra [129]. PPO activity was assessed in a reaction mixture comprising 125 µmol phosphate buffer (pH 6.8), 100 µmol pyrogallol, and 2 mL of enzyme extract. The reaction proceeded for 5 min at 25 °C and then stopped by adding 1 mL of 5% H₂SO₄. A control was prepared with heat-inactivated enzyme extract. The resulting colorimetric change was quantified by measuring the absorbance at 430 nm. All enzyme activities are presented as U/g FW. Also, Total soluble protein content was quantified using the method of Lowry et al. [130].

### **Photosynthetic pigments**

To quantify the concentrations of chlorophyll a, chlorophyll b, and total carotenoids, 0.25 g of fresh weight from the harvested plant leaves was weighed and crushed in absolute acetone using a mortar and pestle until a homogenous mixture formed. The mixture was then refrigerated overnight at 4 °C. Subsequently, the mixture was centrifuged for 10 min at 10,000 × g, and the supernatant was collected and transferred to clean, dark bottles. This procedure was repeated several times until complete extraction of the photosynthetic pigments was achieved. Absorbance measurements were taken at 645 nm (A645), 662 nm (A662), and 470 nm (A470) using a UV/VIS spectrophotometer (RIGOL, MODEL ULTRA-3660). The following equations were used to quantify the contents (µg/mL) of chlorophyll a, chlorophyll b, and total carotenoids [131].

Chlorophyll a = 11.75 A662–2.350 A645.

Chlorophyll b = 18.61 A645–3.960 A662.

Carotene = 1000 A470–2.270 Chl a– 81.4 Chl b/227.

### **Carbohydrate content**

To determine the total carbohydrate content, 0.05 g of dry weight plant leaves were weighed and ground in distilled water with a mortar and pestle to form a homogenous mixture. The mixture was centrifuged at 10,000 × g for 10 min, and the supernatant was collected to measure soluble sugars. The remaining tissue was then used to extract insoluble sugars via (acid hydrolysis) (1% sulfuric acid) in a water bath for 1 h. Following cooling and centrifugation, the supernatant was collected and neutralized with solid sodium carbonate before being transferred to clean bottles. Utilizing the protocol of Dubois et al. [132]., 1 mL of the supernatant was mixed with 5% (w/v) phenol and concentrated sulfuric acid, and the absorbance was measured at 490 nm via a UV‒Vis spectrophotometer. The carbohydrate level was quantified in milligrams per gram of dry weight (mg/g DW), and different concentrations of glucose (0.1-1 mg/mL) were used to create the standard curve where R² value = 0.95.

### **Total phenolic and flavonoid contents**

Total phenolic compounds in maize leaves were analyzed spectrophotometrically at 750 nm following the procedure outlined by Malik and Singh [133]. Briefly, 0.5 mL of leaf extract was mixed with Folin–Ciocalteu reagent and 20% (w/v) sodium carbonate (Na₂CO₃). TPC was expressed as gallic acid equivalent (mg GAE/g DW), based on a standard curve prepared using gallic acid (25–150 µg/mL) where the R² value equaled 0.972. Total flavonoids were measured according to the colorimetric method described by Ali et al. [96]. with minor modifications. The methanolic extract of the sample (0.05 g DW of maize leaves) was mixed with 5% (w/v) sodium nitrite (NaNO_2_), 10% (w/v) aluminum chloride (AlCl_3_), and 1 N sodium hydroxide (NaOH). The reaction was allowed to proceed for 10 min under ambient conditions, and the absorbance was measured at 510 nm. Quercetin served as a standard for the assay with concentrations ranging from 0.1 to 1 mg/mL, where the R² value equaled 0.997. Flavonoid concentrations in the test samples were determined from this plot and expressed as milligrams of quercetin equivalent (QE) per gram of sample (mg QE/g DW) [134].

### **Molecular fingerprinting based on ISSR analysis**

#### **Genomic DNA extraction and ISSR fingerprinting**

Genomic DNA was isolated from young leaves via a DNA extraction kit (Intron Biotechnology, Korea) following the manufacturer’s protocol.

Eight ISSR primers were screened, with 4 producing clear, reproducible polymorphic bands across all species. The name and sequence of the primers are given in Table 7. PCR amplification was carried out in a 20 µL reaction mixture comprising 10 µL of Thermo Scientific Maxima Hot Start PCR Master Mix (2X), 2 µL of primer, 1 µL of template DNA, and 7 µL of distilled water. The thermal cycling conditions consisted of an initial denaturation at 94 °C for 5 min, followed by 35 cycles of denaturation (94 °C, 1 min), annealing (57 °C, 1 min), and extension (72 °C, 1.5 min), with a final extension at 72 °C for 5 min. Amplified products (20 µL per sample) were electrophoresed on a 1% agarose gel, and the banding patterns were visualized, documented, and analyzed via a gel documentation system. All experiments were replicated twice to confirm reproducibility [34].

### **Data analysis**

Each ISSR band was considered an individual genetic locus. Bands were scored as present (1) or absent (0), with polymorphic bands (including unique and nonunique variants) and monomorphic bands recorded. Polymorphisms were evaluated based on band frequency, molecular size, and intensity.

### **Genetic relationship analysis**

Genetic clustering was performed using squared Euclidean distance in PAST-pc Version 4.22 [135], generating a dendrogram to illustrate relationships among accessions. Principal component analysis (PCA) was conducted to produce a scatter diagram [32, 135], while genetic similarity between species was quantified via the Dice similarity coefficient [136]. Estimation of genomic template stability (GTS) revealed genotoxicity, as normal bands disappeared and new bands appeared. Observing only clear and reproducible bands is crucial for assessing DNA order and disorder, as is demonstrating the percentage of genomic template stability (GTS%). The values were calculated for each sample via the formula provided by Sukumaran and Grant [112] as follows:

$$GTS\% = \:\left( {1 - \frac{a}{n}} \right) \times 100,\,\,where$$)

a: average number of polymorphic bands in the samples treated.

n: number of total bands in the control.

The GTS% values were calculated for each sample as the loss/gain of bands, assuming that the GTS% of the control was 100%.

### **Statistical analysis**

The morphological and biochemical parameters were analyzed via one-way analysis of variance (ANOVA) using the Statistical Package for the Social Sciences (SPSS) version 14. Data are presented as means ± standard error (SE), based on three replicate measurements. Significant differences between means were determined using Duncan’s multiple range test (DMRT) at a significance level of *p* < 0.05. The effects of drought, *R. arrhizus* inoculation, and their interactions on several growth, metabolic parameters, and stress markers were further elucidated through two-way ANOVA. The UPGAMA cluster indicated by the program PAST software [135] utilized principal component analysis (PCA), and the color Pearson correlation among various morpho-physiological parameters of the maize plants under the different treatments effectively illustrated the degree of similarity among these parameters on the basis of the Dice coefficient, utilizing the R Studio interface and R software version 4.2.1 (R Studio Team [137]; R Core Team [138]).

## Conclusion

Based on the analysis conducted in this study, it can be demonstrated that the endophytic fungus *Rhizopus arrhizus* significantly enhances drought tolerance in *Zea mays* plants through various mechanisms. The observed results showed enhanced antioxidant enzyme activity, effective osmotic adjustment via increased proline and soluble protein accumulation, mitigation of oxidative stress as evidenced by reduced H₂O₂ and MDA levels, and a continuous regulation of photosynthetic pigment synthesis under drought conditions. Furthermore, *R. arrhizus* colonization promoted carbohydrate, total phenolic, and total flavonoid production, contributing to overall plant resilience under both well-watered and drought-stressed conditions. Notably, the ISSR study showed that *R. arrhizus* inoculation increases genetic template stability in *Zea mays*, indicating a protective function at the genomic level under drought stress. The different genetic profiles seen across treatments demonstrate that *R. arrhizus* reduces genomic changes caused by drought, therefore supporting its possible application in enhancing crop resistance. Genetic template stability (GTS) was notably higher in *Zea mays* plants treated with *Rhizopus* sp. under both control and drought conditions. These results highlight the potential of *R. arrhizus* as a viable bio-inoculant for raising maize tolerance to drought stress, providing a sustainable approach for increasing crop output in water-limited agricultural settings. Future studies should explore the long-term effects of *R. arrhizus* inoculation under field conditions with varying environmental stress levels. Further studies on the molecular mechanisms, especially gene expression related to drought response, antioxidant pathways, and stress-related proteins, can help in understanding the mode of action.

## Electronic supplementary material

Below is the link to the electronic supplementary material.

Supplementary Material 1

## Data Availability

data avaliable upon request.
