# Supplementary material for: Mitigating drought stress and enhancing maize resistance through biopriming with Rhizopus arrhizus: insights into Morpho-Biochemical and molecular adjustments
Source: BMC Plant Biol. 2025 Jun 11;25:779. doi: 10.1186/s12870-025-06793-3 (PMC12160352; doi:10.1186/s12870-025-06793-3)
Supplement: Supplementary file 1 — Supplementary Material 1 [file 12870_2025_6793_MOESM1_ESM.docx]

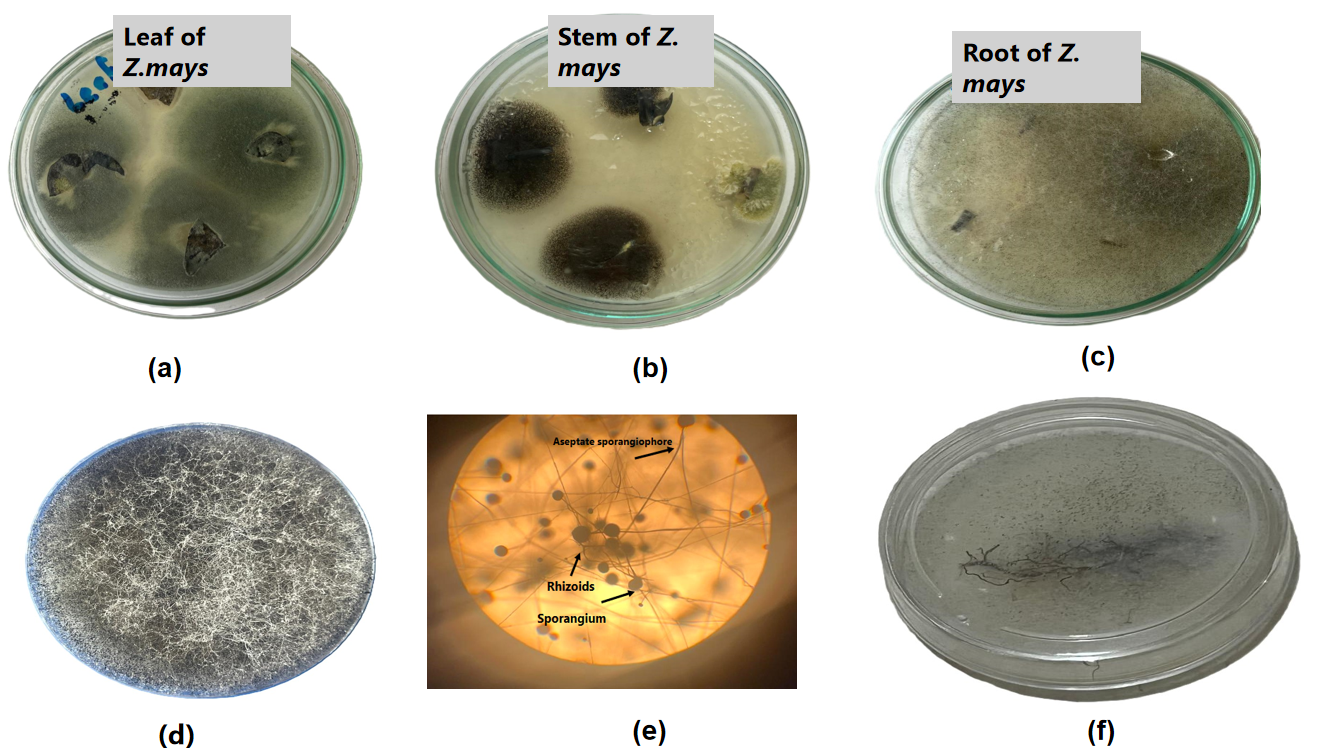


**Fig. 1** Isolation of endophytic fungi from *Z. mays* **(a)** leaves, **(b)** stem, and **(c)** roots. **(d)** the growth of *Rhizopus* on potato dextrose agar (PDA) medium, **(e**) photograph of *Rhizopus* under a light microscope showing sporangium, aseptate sporangiophore, and rhizoids, and **(f)** plating long segments of *Z. mays* root on a PDA plate confirming colonization of roots by *Rhizopus*.

**
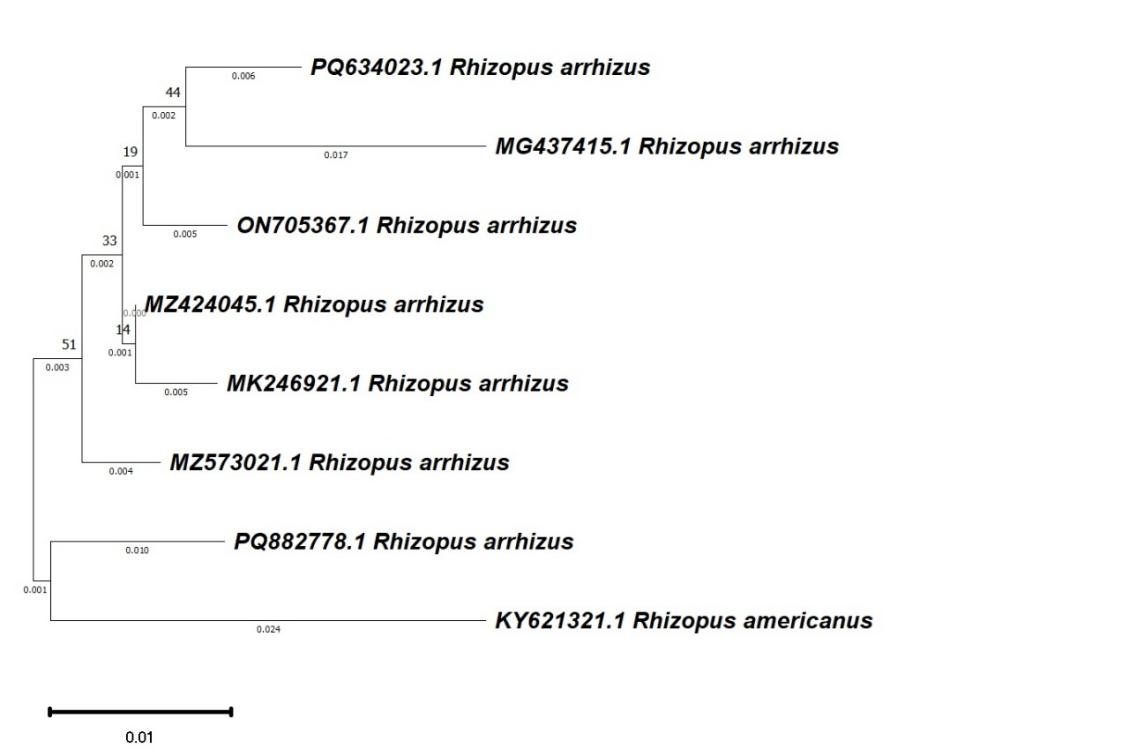
**

**Fig. 2** Phylogenetic analysis of *Rhizopus* *arrhizus* (acc no. PQ882778) demonstrating the ITS connection with closely similar strains' ITS sequences that were retrieved from NCBI GenBank database. In the MEGA7 software, evolutionary analyses were carried out.


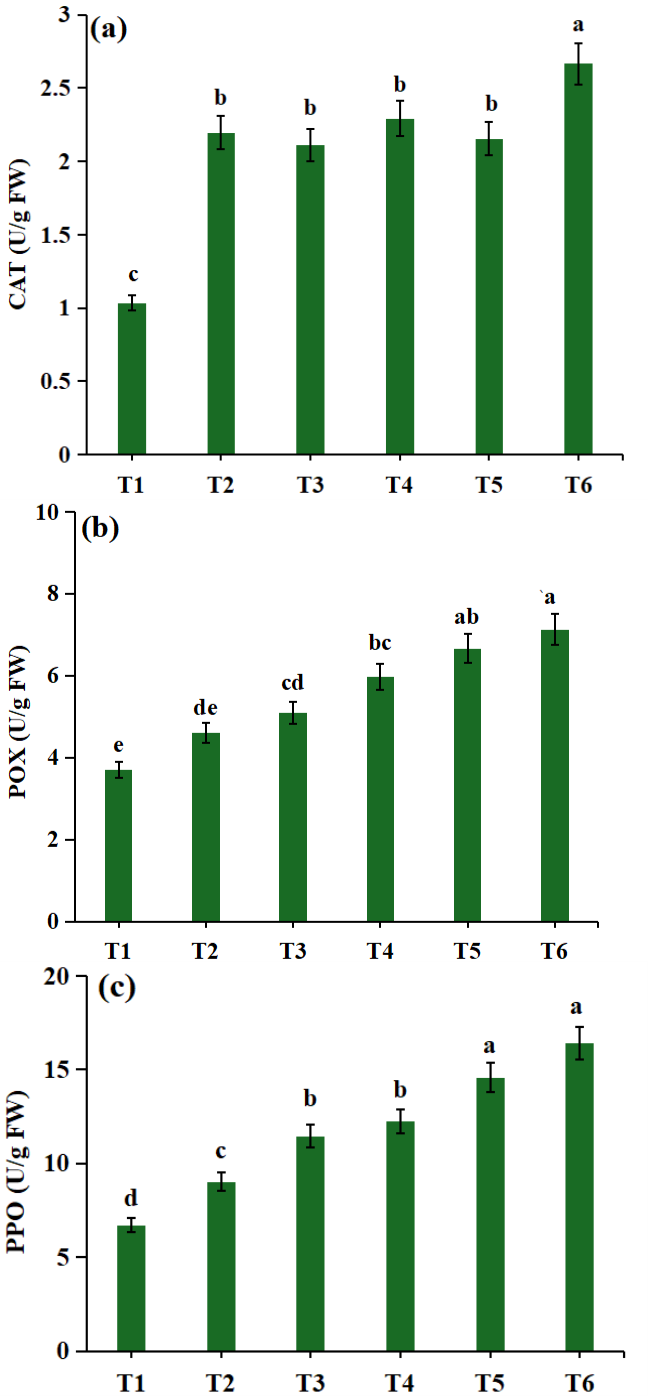


**Fig. 3** Effect of *Rhizopus* application on antioxidant enzymes system: **(a)** CAT, **(b)** POX, and **(c)** PPO in *Zea mays* plants in response to drought stress. Results are the mean of three replicates±SE. Different letters above bars indicate a significant difference between treatments using One-Way ANOVA followed by Duncan’s multiple range test (DMRT) (*p* < 0.05).Treatments: **T1**－control (800 mL H₂O); **T2**－control with *Rhizopus*; **T3**－400 mL H₂O; **T4** －400 mL H₂O with *Rhizopus*; **T5**－200 mL H₂O; **T6**－200 mL H₂O with *Rhizopus*.


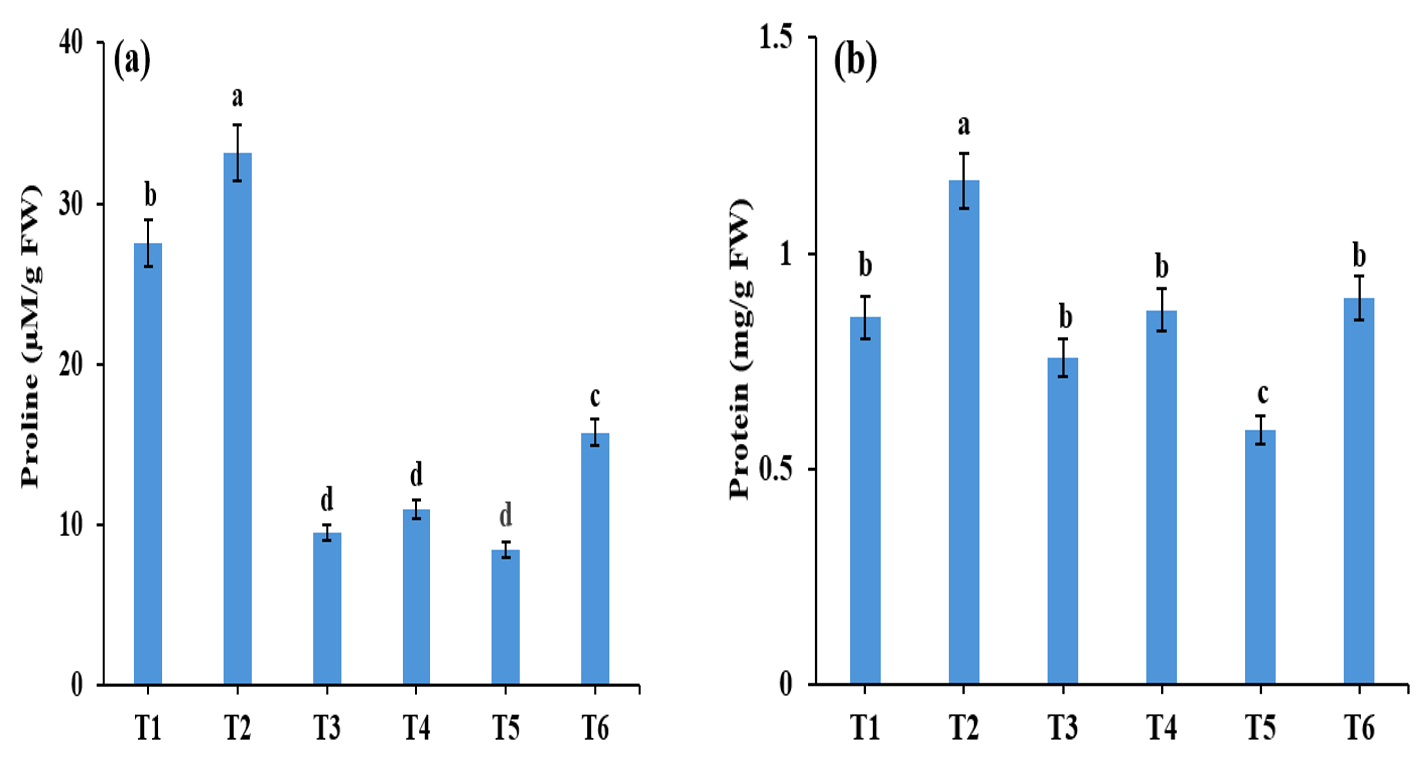


**Fig. 4** Effect of *Rhizopus* application on **(a)** proline and **(b)** total soluble protein in *Zea mays* plants in response to drought stress. Results are the mean of three replicates±SE. Different letters above bars indicate a significant difference between treatments using One-Way ANOVA followed by Duncan’s multiple range test (DMRT) (*p* < 0.05).Treatments: **T1**－control (800 mL H₂O); **T2**－control with *Rhizopus*; **T3**－400 mL H₂O; **T4** －400 mL H₂O with *Rhizopus*; **T5**－200 mL H₂O; **T6**－200 mL H₂O with *Rhizopus*.


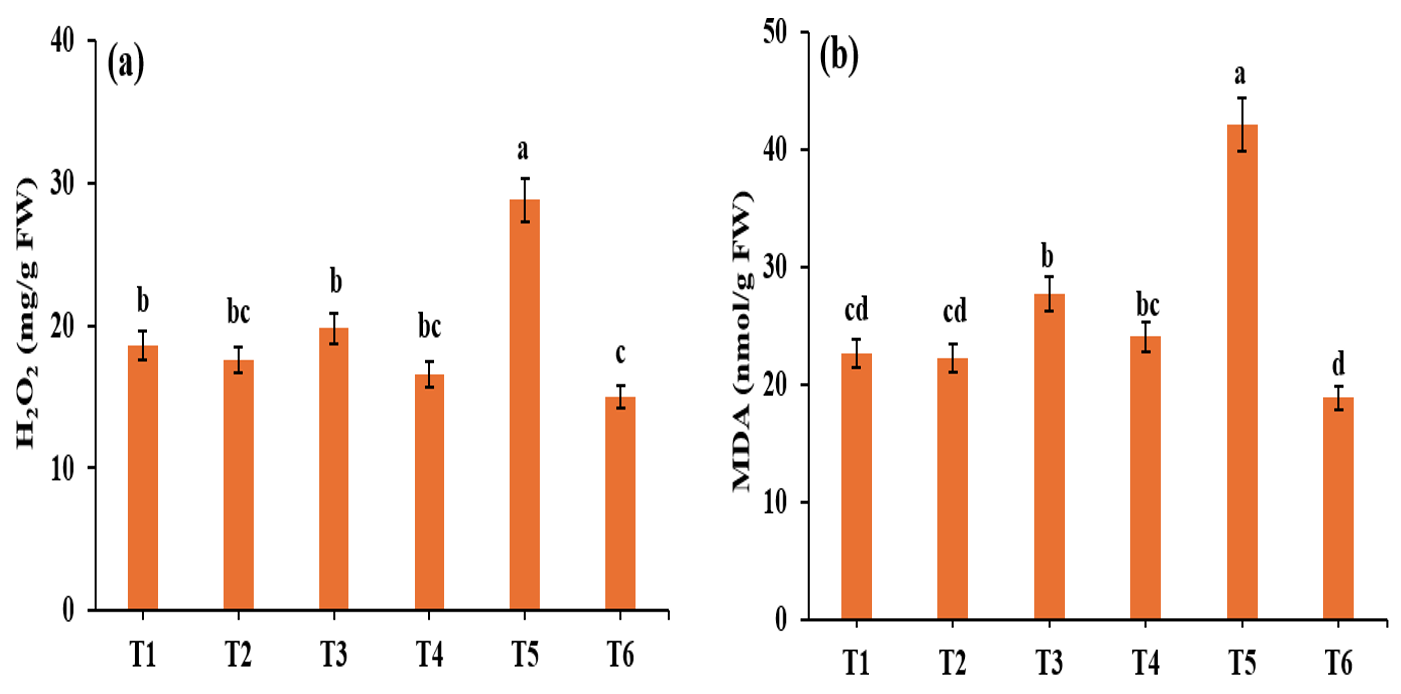


**Fig. 5** Effect of *Rhizopus* application on stress markers: **(a)** H_2_O_2_ and **(b)** malondialdehyde (MDA) in *Zea mays* plants in response to drought stress. Results are the mean of three replicates±SE. Different letters above bars indicate a significant difference between treatments using One-Way ANOVA followed by Duncan’s multiple range test (DMRT) (*p* < 0.05). Treatments: **T1**－control (800 mL H₂O); **T2**－control with *Rhizopus*; **T3**－400 mL H₂O; **T4** －400 mL H₂O with *Rhizopus*; **T5**－200 mL H₂O; **T6**－200 mL H₂O with *Rhizopus*.


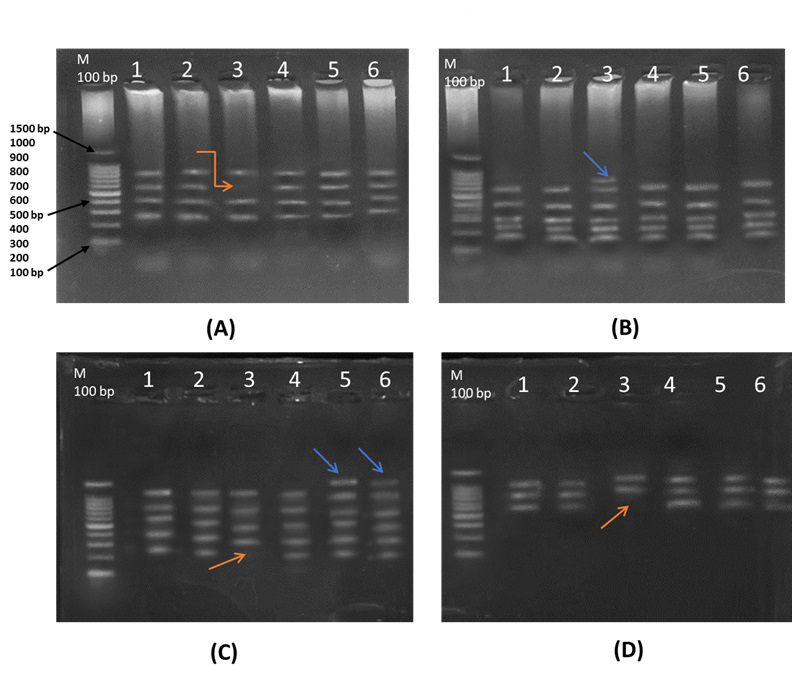


**Fig. 6** Products of genomic DNA using Inter Simple Sequence Repeats (ISSR) extracted from leaves of (*Z. mays*) at different treatments. The lanes referred to germplasm of 6 treatments. Lane M =1.5 Kb DNA marker.Treatments: **T1**－control (800 mL H₂O); **T2**－control with *Rhizopus*; **T3**－400 mL H₂O; **T4** －400 mL H₂O with *Rhizopus*; **T5**－200 mL H₂O; **T6**－200 mL H₂O with *Rhizopus*.


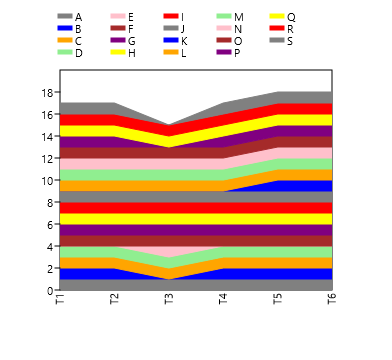


**Fig. 7** A stacked area created with PAST-pc that displays the band number of amplified DNA markers produced by four ISSR primers in the samples under study.Treatments: **T1**－control (800 mL H₂O); **T2**－control with *Rhizopus*; **T3**－400 mL H₂O; **T4** －400 mL H₂O with *Rhizopus*; **T5**－200 mL H₂O; **T6**－200 mL H₂O with *Rhizopus*.


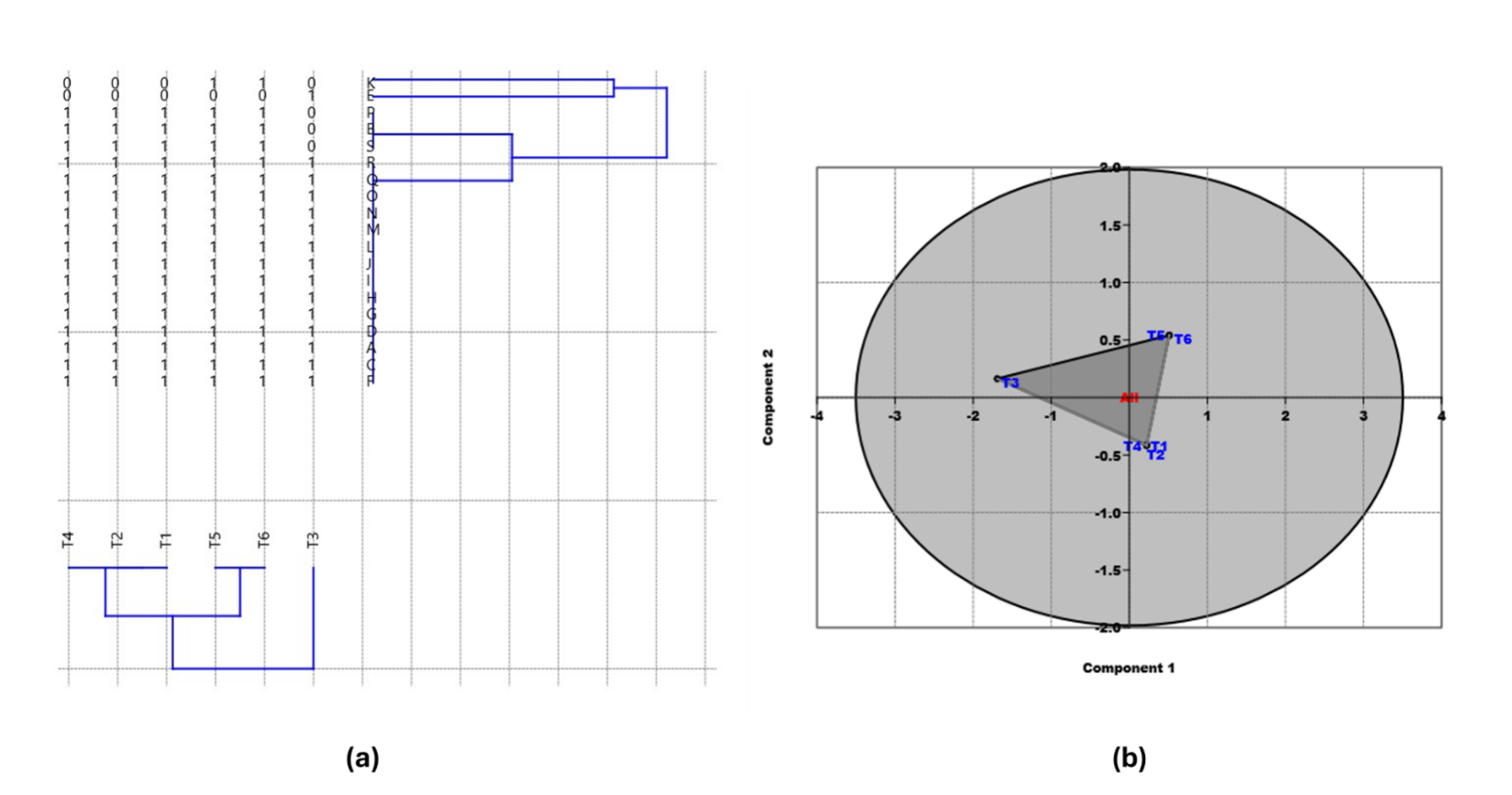


**Fig. 8 (a)** Euclidean-based UPGAMA distance tree built using PAST-pc that displays the genetic separation among various treatments **(b)** PCA built with PAST-pc that displays the band number of amplified DNA markers produced by the ISSR marker.Treatments: **T1**－control (800 mL H₂O); **T2**－control with *Rhizopus*; **T3**－400 mL H₂O; **T4** －400 mL H₂O with *Rhizopus*; **T5**－200 mL H₂O; **T6**－200 mL H₂O with *Rhizopus*.


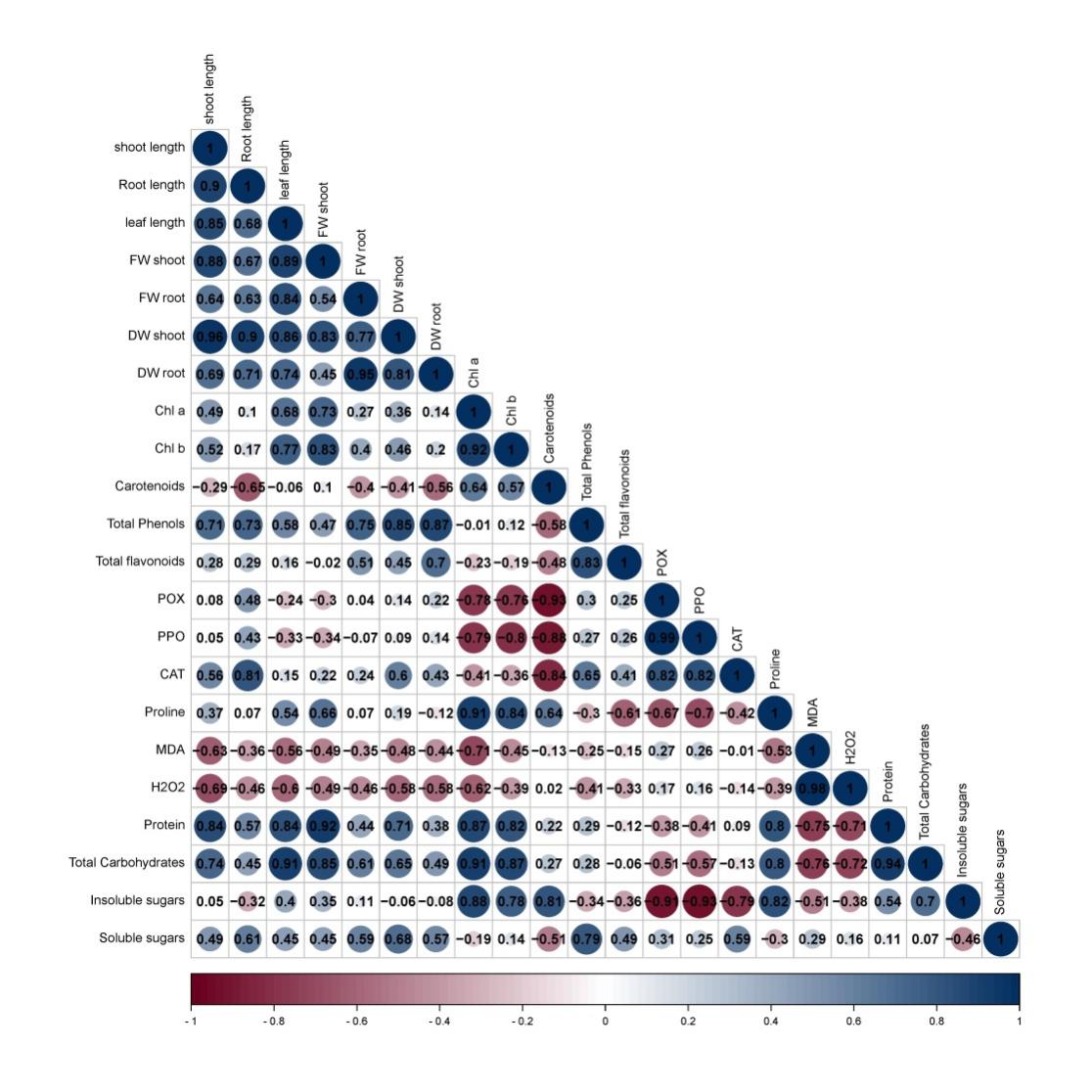


**Fig. 9** Pearson correlation among morphological and biochemical parameters for *Z. mays* plant after treatments. The boxed points correspond with significant correlation at *p* < 0.05 (blue: positive ; red: negative).


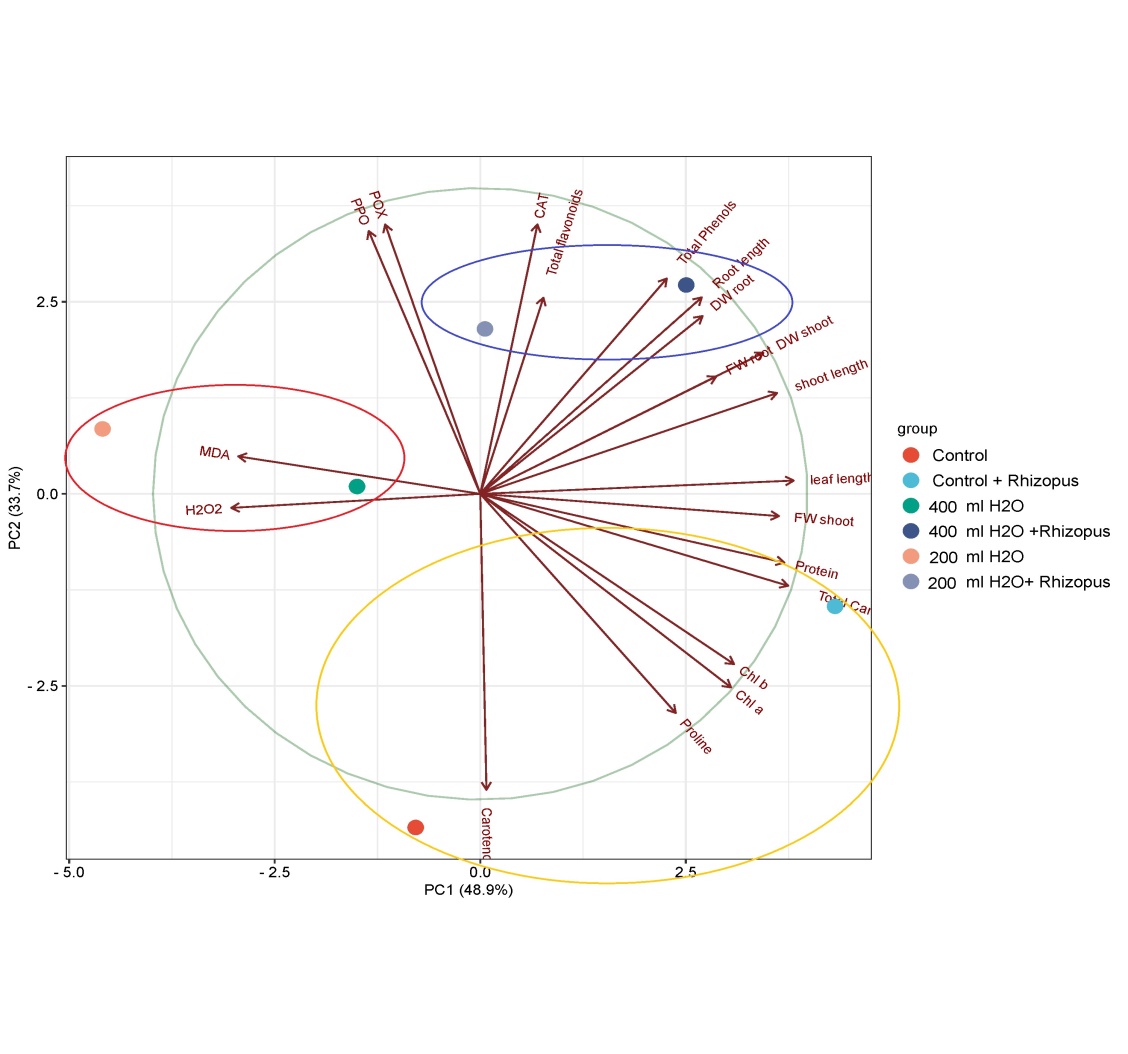


**Fig. 10** Principal component analysis (PCA) among different treatments of *Z. mays* based on morphological and biochemical analyses.
